# Identification of new overlapping and disease-specific genetic risk factors for rheumatoid arthritis and radiographic axial spondyloarthritis: a meta-analysis of three large European populations and functional characterization

**DOI:** 10.3389/fimmu.2026.1637735

**Published:** 2026-04-23

**Authors:** Antonio José Cabrera-Serrano, María Carretero-Fernández, Begoña Pérez-Rojo, Rob ter Horst, Marisa Cañadas-Garre, Helena Canhão, Luca Quartuccio, Signe B. Sorensen, Bente Glintborg, Ileana Filipescu, Eva Pérez-Pampin, Pablo Conesa-Zamora, Jerzy Swierkot, Alfons A. den Broeder, Salvatore de Vita, Eva Rabing Brix Petersen, Yang Li, Marieke J. H. Coenen, Katarzyna Bogunia-Kubik, Vibeke Andersen, João Eurico Fonseca, Merete Lund Hetland, Miguel Ángel López Nevot, Clementina López-Medina, Fernando Jesús Reyes-Zurita, Mihai G. Netea, Alejandro Escudero, Rafael Cáliz, Eduardo Collantes-Estévez, José Manuel Sánchez-Maldonado, Juan Sainz

**Affiliations:** 1 Genomic Oncology Area, GENYO, Centre for Genomics and Oncological Research: Pfizer/University of Granada/Andalusian Regional Government, PTS Granada, Granada, Spain; 2 Instituto de Investigación Biosanitaria IBs.Granada, Granada, Spain; 3 Department of Internal Medicine and Radboud Center for Infectious Diseases, Radboud University Nijmegen Medical Center, Nijmegen, Netherlands; 4 CeMM Research Center for Molecular Medicine of the Austrian Academy of Sciences, Vienna, Austria; 5 Medical University of Vienna, Center for Medical Data Science, Institute of Artificial Intelligence, Vienna, Austria; 6 EpiDoC Unit, CEDOC, NOVA Medical School and National School of Public Health, Universidade Nova de Lisboa, Lisbon, Portugal; 7 Comprehensive Health Research Center (CHRC), NOVA Medical School, Lisbon, Portugal; 8 Department of Medical Area, Clinic of Rheumatology, University of Udine, Udine, Italy; 9 Molecular Diagnostic and Clinical Research Unit, IRS-Center Sonderjylland, University Hospital of Southern Jutland, Aabenraa, Denmark; 10 Institute of Molecular Medicine, Faculty of Health Sciences, University of Southern Denmark, Odense, Denmark; 11 The DANBIO Registry, The Danish Rheumatologic Biobank and Copenhagen Center for Arthritis Research (COPECARE), Center for Rheumatology and Spine Diseases, Centre of Head and Orthopaedics, Rigshospitalet, Glostrup, Denmark; 12 Department of Clinical Medicine, Faculty of Health and Medical Sciences, University of Copenhagen, Copenhagen, Denmark; 13 Rheumatology Department, University of Medicine and Pharmacy “Iuliu Hatieganu” Cluj-Napoca, Cluj-Napoca, Romania; 14 Rheumatology Unit, University Hospital of Santiago de Compostela, Santiago de Compostela, Spain; 15 Clinical Analysis Department, Santa Lucía University Hospital, Cartagena, Spain; 16 Department of Rheumatology and Internal Medicine, Wroclaw Medical University, Wroclaw, Poland; 17 Department of Rheumatology, Radboud University Medical Center, Radboud Institute for Health Sciences, Nijmegen, Netherlands; 18 Department of Biochemistry and Immunology. University Hospital of Southern Jutland, Aabenraa, Denmark; 19 Centre for Individualised Infection Medicine (CiiM) and TWINCORE, joint ventures between the Helmholtz-Centre for Infection Research (HZI) and the Hannover Medical School (MHH), Hannover, Germany; 20 Department of Human Genetics, Radboud University Medical Center, Radboud Institute for Health Sciences, Nijmegen, Netherlands; 21 Hirszfeld Institute of Immunology and Experimental Therapy, Polish Academy of Sciences, Wrocław, Poland; 22 Institute of Regional Research, Faculty of Health Sciences, University of Southern Denmark, Odense, Denmark; 23 Rheumatology and Metabolic Bone Diseases Department, Hospital de Santa Maria, CHLN, Lisbon, Portugal; 24 Rheumatology Research Unit, Instituto de Medicina Molecular, Faculty of Medicine, University of Lisbon, Lisbon Academic Medical Center, Lisbon, Portugal; 25 Immunology Department, Virgen de las Nieves University Hospital, Granada, Spain; 26 Rheumatology Department, Reina Sofía Hospital/IMIBIC/University of Córdoba, Córdoba, Spain; 27 Department of Biochemistry and Molecular Biology, Faculty of Sciences, University of Granada, Granada, Spain; 28 Department for Immunology and Metabolism, Life and Medical Sciences Institute (LIMES), University of Bonn, Bonn, Germany; 29 Rheumatology Department, Virgen de las Nieves University Hospital, Granada, Spain; 30 Consortium for Biomedical Research in Epidemiology and Public Health (CIBERESP), Barcelona, Spain

**Keywords:** rheumatoid arthritis, ankylosing spondylitis, genetic variants, overlapping and disease-specific genetic markers, functional characterization

## Abstract

**Introduction:**

This study conducted a meta-analysis across three large European cohorts (UKBB, FinnGen, and REPAIR), including 12,660 rheumatoid arthritis (RA) cases, 2,446 radiographic axial spondyloarthritis (r-axSpA) cases, and over 530,000 shared controls.

**Methods:**

Ten independent SNPs in *CARMIL1*, *GRM4*, *ITPR3*, *PRSS16*, *ZNF322*, *HTT*, *IKZF1*, *MANEA*, and *MGAM2* were analyzed, and functional characterization was performed through cytokine and protein assessments as well as eQTL analyses.

**Results:**

Ten independent SNPs were significantly associated with both RA and r-axSpA. Risk alleles included *HTT*_rs363075A_, *IKZF1*_rs12718261A_, *MANEA*_rs72920280T_, and *MGAM2*_rs73158426G_, while *CARMIL1*_rs72831267C_, *GRM4*_rs2495964G_, *ITPR3*_rs77601296A_, *ITPR3*_rs9469540T_, *PRSS16*_rs72843633T_, and *ZNF322*_rs6901425G_ had protective effects. Functional analysis showed that *GRM4*_rs2495964G_ was linked to decreased CCL25 levels (p = 0.00030), and *ITPR3*_rs9469540T_ to reduced IL10 production after LPS stimulation (p = 1.3×10^−4^). The ZNF322rs6901425G allele was associated with reduced TNFB and increased TGM2 levels (p = 9.60×10^−4^ and p = 3.00×^10−4^), both involved in immune signaling and tissue remodeling. Disease-specific associations were found in *BTN2A1*, *BTN3A2*, and *H2BC11*. The *BTN2A1*_rs1977199A_ allele was protective in RA (OR = 0.93) but increased r-axSpA risk (OR = 1.23), and was associated with reduced IL22 (p = 0.00016) and elevated HO-1 in obese individuals (p = 6.73×10^−6^). In contrast, *BTN3A2*_rs9393716G_ and *H2BC11*_rs66462181C_ increased RA risk but were protective in r-axSpA, linked to decreased HO-1 and IL6 (p = 2.43×10^−5^, 3.287times;10^−4^, 1.18×10^−4^). These SNPs also acted as eQTLs for immune-related genes such as *BTN3A2*, *HMGN4*, and *TRIM38*.

**Discussion:**

Our findings highlight novel shared and disease-specific variants and key immunoregulatory mediators—IL10, IL22, IL6, CCL25, and HO-1—offering insights for disease stratification and therapeutic targeting.

## Introduction

Rheumatoid Arthritis (RA) is a complex and chronic immune-mediated inflammatory arthritis characterized by joint pain and swelling leading to disability and joint destruction (1). It is broadly known that RA predisposition is partially explained by common genetic variants within multiple immune-related loci, including class I-II human leukocyte antigen (HLA) genes (*HLA-A*, *HLA-B*, *HLA-S*, *HLA-DR1*, *HLA-DR4*, *HLA-DQA1*), but also genes such as *CD40*, *CCL21*, *CCR6, IL2RA*, *IL2RB*, *IL23, IRF5*, *PADI4*, *PTPN22*, *STAT4*, *TRAF1*/*C5*, and *TYK2* (2–9). Genome-wide association studies (GWAS) have consistently demonstrated that RA shares multiple susceptibility loci with other immune-mediated inflammatory diseases (IMIDs) including radiographic axial spondyloarthritis (r-axSpA), but also type 1 diabetes (T1D), inflammatory bowel disease (IBD), celiac disease (CD), psoriasis (Ps), and autoimmune thyroid disease (AITD) (10–15). Although RA and r-axSpA are distinct in their clinical features, age of onset, and sex distribution, understanding potential overlapping factors between them remains important. Notably, HLA-B27 is closely linked to r-axSpA, whereas it is not typically associated with RA. Nevertheless, both diseases share comparable incidence rates, ranging from 0.3% to 1.0% (16), and certain manifestations in RA—such as cervical spine involvement (17)—can have serious neurological and structural consequences, including degenerative myelopathy and increased mortality (18). Furthermore, although uncommon, several studies have reported cases in which RA and r-axSpA may coexist in the same individual, suggesting that overlapping etiological factors may occasionally play a role (19). Exploring shared genetic, inflammatory, or structural pathways may help uncover common mechanisms underlying joint and spinal damage, especially as advances in diagnostic and laboratory technologies continue to refine disease classification (20).

Considering this background, the aim of this study was to identify new overlapping and disease-specific susceptibility variants for RA and r-axSpA and to functionally characterize their impact on the onset of both diseases. For that purpose, we analyzed association estimates for RA and r-axSpA in the UK Biobank (UKBB) and FinnGen cohorts, and we validated the association of the most relevant overlapping and disease-specific susceptibility variants in RA and r-axSpA populations from the RhEumatoid Arthritis International Research consortium (REPAIR) consortium. We also investigated whether the effect of the overlapping and disease-specific markers could influence host immune responses *in vitro* and determine the absolute numbers of 91 blood-derived immune cell populations and circulating concentrations of 106 plasmatic inflammatory proteins and 7 serum steroid hormones in the 500 Functional Genomics (500FG) and 300 Obesity (300OB) cohorts from the Human Functional Genomics Project (HFGP).

## Materials and methods

### Discovery populations and phenotype definitions

The discovery cohorts consisted of two large populations of European ancestry ascertained through the UK Biobank (UKBB) (project ID: 24460; available at PheWeb: https://pheweb.sph.umich.edu) (21, 22) and FinnGen projects (freeze R5, released in March 2020; https://finngen.gitbook.io/documentation/). Endpoint definitions for the UKBB were generated from electronic health records derived from International Classification of Diseases (ICD) billing codes, whereas those for the FinnGen data are available at https://risteys.finregistry.fi/.

Disease phenotypes were defined using clinically curated electronic health record–based endpoints rather than self-reported diagnoses. In the UKBB, rheumatoid arthritis cases were identified using hospital episode statistics and ICD-10 codes (M05–M06). Radiographic axial spondyloarthritis (r-axSpA) cases were defined using ICD-10 diagnostic codes corresponding to ankylosing spondylitis and related spondyloarthropathies (including M45), following the phenotype definitions provided by the UK Biobank consortium.

In FinnGen, case definitions were based on national registry data integrating hospital discharge diagnoses, outpatient specialist visits, and reimbursement records, using validated ICD-based endpoints for both RA and r-axSpA. Controls were defined as individuals without recorded diagnoses of inflammatory arthritis.

For RA, a total of 4,380 cases and 363,562 controls were included from the Trans-Omics for Precision Medicine (TOPMed)-imputed dataset of the UKBB (https://pheweb.org/UKB-TOPMed/pheno/714.1), and 6,236 RA cases and 147,221 controls from FinnGen (https://r5.finngen.fi/pheno/M13_RHEUMA). Additionally, 617 r-axSpA cases and 363,562 controls were available from the UKBB using the same imputed panel (https://pheweb.org/UKB-TOPMed/pheno/715.2) and 1,462 r-axSpA cases and 164,682 controls were included from FinnGen (https://r5.finngen.fi/pheno/M13_ANKYLOSPON) (**Table 1**).

**Table 1:** Demographic and clinical characteristics of the discovery and replication populations.

Discovery populations							
RA cohort				AS cohort			
	Cases	Controls	N		Cases	Controls	N
UKBB	4,380	363,562	367,942	UKBB	617	363,562	364,179
FinnGen	6,236	147,221	153,457	FinnGen	1,462	164,682	166,144
Replication populations (REPAIR cohorts)							
RA cohort				AS cohort			
	Cases	Controls	N		Cases	Controls	N
Spain	638	954	1,592	Spain	274	954	1,228
Portugal	708	176	884	Portugal	-	176	176
Denmark	583	785	1,368	Denmark	-	785	785
Italy	35	24	59	Italy	-	24	24
Rumania	80	96	176	Rumania	-	96	96
Poland	-	-	-	Poland	93	115	208
Total	2,044	2,035	4,079	Total	367	2,150	2,517
Age	59.85 ± 14.61	49.05 ± 10.62	54.17 ± 13.77	Age	33.88 ± 10.68	48.48 ± 10.89)	46.05 ± 12.14
Males	344 (21.81%)	751 (43.29%)	1,095 (33.06%)	Males	281 (76.57%)	793 (42.86%)	1,074 (48.44%)
Females	1.233 (78.19%)	984 (56.71%)	2.217 (66.94%)	Females	86 (23.43%)	1,057 (57.14%)	1,143 (51.56%)

Although the prevalence of r-axSpA is lower than that of RA in population-based biobanks, this limitation is inherent to registry-based genetic studies of spondyloarthritis and is partially mitigated by the large combined sample size across cohorts.

### Ancestry and population structure

All analyses were restricted to participants of European ancestry, as defined by cohort-specific genetic principal component–based ancestry assignments provided in the original GWAS summary statistics. This restriction was applied to minimize population stratification and ensure comparability across datasets used in the meta-analysis. Although the Finnish population represents a genetic isolate with specific allele frequency characteristics, it is classified within the broader European ancestry group and was therefore included in the European-only analytical framework. This approach is consistent with previous large-scale genetic meta-analyses and avoids introducing continental-level ancestry heterogeneity (23).

### Genetic association analysis in the discovery cohorts and meta-analysis

A two-stage analytical framework was applied. In the first stage, SNPs jointly associated with both RA and r-axSpA were selected using a suggestive significance threshold (p < 1×10^−3^) to capture shared genetic signals and reduce false-negative discovery of overlapping risk variants. This threshold was applied as a pre-filtering step to identify shared signals and should not be interpreted as genome-wide significance. In the second stage, overlapping variants were further prioritized using functional annotation, regulatory enrichment, and expression quantitative trait locus (eQTL) analyses. The Bonferroni method was used to account for multiple testing, and a p-value of p < 3.33 × 10^−3^ (0.05/15 independent SNPs) was set as the study-wide significance threshold.

The association estimates of these respective GWAS were then meta-analyzed using METAL (24), and heterogeneity across studies was evaluated using the I² statistic. SNPs showing consistent effect directions and no significant heterogeneity were retained for downstream replication analyses. Given the strong immunogenetic relevance of the extended MHC region (chr6:29–34 Mb) for both RA and r-axSpA, this region was retained in the primary analysis to capture shared immune-related genetic architecture. To minimize potential bias due to complex linkage disequilibrium patterns, independent association signals were defined using stringent LD clumping criteria and heterogeneity filtering across cohorts. This two-stage prioritization framework is consistent with pleiotropy-informed GWAS approaches, which demonstrate that leveraging cross-phenotype information improves power to detect shared loci and support the use of permissive initial filters followed by rigorous downstream statistical control, particularly when phenotypes differ in statistical power. To further ensure that retained variants represented independent association signals, linkage disequilibrium (LD) between SNPs was calculated (r^2^ = 0.60 and a window size of 500Kb) using the 1000 Genomes phase 3 (reference build 37; https://ftp.1000genomes.ebi.ac.uk/vol1/ftp/release/20130502/) as the reference panel. To further ensure that prioritized signals were not driven by classical HLA effects, additional LD analyses were performed between prioritized chromosome 6 lead variants and established proxies of classical HLA risk alleles (including markers tagging HLA-B27 and HLA-DRB1) using European populations from the 1000 Genomes Project (see **Supplementary Table 1**).

### Replication populations

For validation purposes, we included a third European population ascertained through the REPAIR consortium, which included 2,411 IMID patients (2,044 RA patients and 367 r-axSpA cases) and 2,150 healthy controls (**Table 1**). The REPAIR study followed the Declaration of Helsinki. Study participants were of European origin and gave their written informed consent to participate in the study, which was approved by the ethical review committee of all participant institutions: Virgen de las Nieves University Hospital (2012/89); Santa Maria Hospital-CHLN (CE 877/121.2012); University Clinical Hospital of Santiago de Compostela (2013/156); Wroclaw Medical University (KB-625/2016); and by the Radboud university medical center (2011/299). A detailed description of the REPAIR population has been reported elsewhere (25). RA and r-axSpA patients fulfilled the ACR/EULAR 2010 classification criteria (26).

### DNA extraction and genotyping in the validation cohort

Genomic DNA from RA and r-axSpA patients was extracted from blood samples using the QIAamp DNA Blood Mini kit (Qiagen Valencia, CA, USA) according to the manufacturer’s instructions. Genotyping of the independent SNPs selected for replication in the REPAIR cohort was carried out at GENYO (PTS Granada, Spain) using KASPar^®^ (LGC Genomics, Hoddesdon, UK) or Taqman^®^ SNP Genotyping assays (Thermo Fisher Scientific, Foster City, CA, USA) according to previously reported protocols. For internal quality control, approximately 5% of samples were randomly duplicated; concordance between original and duplicate genotypes was ≥99.0%. All SNPs showed genotype frequencies in the control population consistent with those in the 1000 Genomes database and were in Hardy-Weinberg equilibrium (HWE) (*p* < 10^-3^).

### Statistical analysis in the validation cohorts and global meta-analysis

The HWE test was performed in the control group using a standard chi-square (χ^2^) test. Logistic regression analyses, adjusted for age, gender, and country of origin, were used to assess the effects of genetic polymorphisms on RA and r-axSpA risk using a log-additive model. All analyses were conducted using STATA (version 20.0). Subsequently, to validate the most interesting shared and disease-specific associations for RA and r-axSpA, a meta-analysis of the discovery populations (UKBB and FinnGen (**Supplementary Table 2**) and replication REPAIR (**Supplementary Table 3**) cohorts was conducted using METAL. As before, the I^2^ statistics were used to assess statistical heterogeneity among the three cohorts. The pooled odds ratio (OR) was computed using a fixed-effect model. The Bonferroni method was used to account for multiple testing, and a p-value of *p* < 3.33×10^-3^ (0.05/15 independent SNPs) was set as the study-wide significance threshold.

### Correlation between overlapping markers and cytokine quantitative trait loci and hormone analyses

With the aim of determining whether the overlapping SNPs, or those SNPs inversely associated with RA and r-axSpA, had an effect on modulating host immune responses, we performed *in vitro* stimulation experiments and measured cytokine production (IFNγ, IL1Ra, IL1β, IL6, IL8, IL10, TNFα, IL17, and IL22) after stimulation of peripheral blood mononuclear cells (PBMCs), whole blood (WB) or monocyte-derived macrophages (MDMs) with lipopolysaccharide (LPS; 1 or 100 ng/ml), phytohaemagglutinin (PHA; 10μg/ml), Pam3Cys (10μg/ml), CpG oligodeoxynucleotide (ODN M362; 10μg/ml), *Escherichia coli*, and *Staphylococcus aureus*. Stimulation experiments were conducted in 408 healthy subjects and 302 obese/overweighted individuals of the 500FG and 300OB cohorts of the HFGP following previously described protocols (27, 28).

Given the influence of sex and steroid hormones on immune responses and disease course, we also evaluated correlations between selected SNPs and circulating levels of seven steroid hormones (androstenedione, cortisol, 11-deoxy-cortisol, 17- hydroxy-progesterone, progesterone, testosterone and 25-hydroxy vitamin D3) in a subset of the 500FG cohort (n=279), excluding individuals undergoing hormonal replacement therapy or taking oral contraceptives. After logarithmic transformation, cytokine or serum steroid hormone levels were correlated with SNPs using linear regression with age and sex as covariates in R software (http://www.r-project.org/). This generated cytokine quantitative trait loci (cQTL) and hormone quantitative trait loci (hQTL). Considering 15 SNPs and 9 cytokines analyzed in the stimulation experiments, the significance threshold for cQTL analysis was *p* = 3.70×10^-4^; for hQTL (15 SNPs x 7 hormones), *p* = 4.76×10^-4^.

### Correlation between overlapping markers and blood-derived cell populations and inflammatory proteins

We also investigated whether selected polymorphisms had an impact on blood cell counts by analyzing a set of 91 manually annotated immune cell populations and genotype data from the 500FG cohort (**Supplementary Table 4**). Cell populations were measured using 10-color flow cytometry (Navios flow cytometer, Beckman Coulter) within 2–3 hours after blood sampling, and data were processed with Kaluza software (v. 1.3, Beckman Coulter). To reduce inter-experimental noise and increase statistical power, cell count analyses were based on parental and grandparental percentages, which were defined as the percentage of a certain cell type within the cell populations one or two levels higher in the hierarchical definitions of cell sub-populations (28). Detailed laboratory protocols for cell isolation, reagents, gating, and flow cytometry analysis are reported elsewhere (29), and flow cytometry data are available upon request (http://hfgp.bbmri.nl).

Serum and plasma proteomics were assessed in the 500FG cohort using the Olink^®^ Inflammation panel (Olink, Sweden), quantifying 103 biomarkers (**Supplementary Table 5**). Protein concentrations were expressed as log_2_-transformed normalized protein expression (NPX) values and further normalized using bridging samples to correct for batch effects (30). Considering the number of proteins (n=103) and cell populations (n=91) tested, significant thresholds were set at *p* = 3.23×10^-5^ (0.05/15 SNPs/103 inflammatory proteins) and *p* = 3.66×10^-5^ (0.05/15 SNPs/91 blood cell types), respectively.

### *In silico* characterization of overlapping and disease-specific genetic markers

To further investigate the functional effect of the most relevant overlapping and disease-specific SNPs, Haploreg (http://www.broadinstitute.org/mammals/haploreg/haploreg.php, accessed on 6 February 2025) and ENCODE (Encyclopedia of DNA Elements) annotation data (https://genome.ucsc.edu/ENCODE) were used to predict their functional roles. Finally, we evaluated whether overlapping and disease-specific SNPs acted as expression quantitative trait loci (eQTL) across different cell types and tissues using data from the Genotype-Tissue Expression (GTex) portal (V8 release, https://gtexportal.org/home/, accessed 20 January 2025).

## Results

The discovery populations included 12,695 IMID patients (10,616 RA cases and 2,079 r-axSpA cases) and 510,783 or 528,244 controls from the UKBB and FinnGen GWAS datasets. All SNPs analyzed showed no deviation from HWE (*p* < 1×10^-3^) in either dataset.

A total of 10,468,350 variants were included in the meta-analysis of both datasets. After filtering by consistent directions of the β effect and ensuring a lack of heterogeneity between studies (*P_Het_*≥0.05), 3,702 variants were associated with RA or r-axSpA risk at *p* < 1×10^-3^. After LD clumping (r^2^ = 0.10, 500-kb window), 15 LD blocks within 14 genes were associated with RA risk and 15 LD blocks within 15 genes with r-axSpA risk (**Table 2**). Importantly, several prioritized variants reached genome-wide significance in disease-specific meta-analyses (e.g., *ITPR3*, *BTN2A1* and *BTN3A2*), supporting the robustness of the shared loci identified. These SNPs were located outside of the HLA region and were not in LD (r^2^ <0.2) with genetic variants previously identified through GWAS, indicating potentially novel associations.

**Table 2:** Selected SNPs.

SNP	Nearest genes	Chr.	Pos. (GRCh38.p7)	Effect allele	SNP location
rs363075	*HTT*	4	3135947	A	Intronic
rs1977199	*BTN2A1*	6	26466161	A	Intronic
rs6901425	*ZNF322*	6	26670418	G	Intronic
rs9393716	*BTN3A2*	6	26376640	G	Intronic
rs12718261	*IKZF1*	7	50290310	A	Intronic
rs66462181	*H2BC11*	6	27123882	C	Near promoter
rs72831267	*CARMIL1*	6	25436727	C	Intronic
rs72843633	*PRSS16*	6	27215170	T	Intronic
rs72920280	*MANEA*	6	94957226	T	Intronic
rs73158426	*MGAM2*	7	142118538	G	Intronic
rs77601296	*ITPR3*	6	33633118	A	Intronic
rs2495964	*GRM4*	6	33951273	G	Intronic
rs9469540	*ITPR3*	6	33652143	T	Intronic
rs71559061	*ERAP2*	6	28012011	G	Intronic
rs72880049	*FTO*	6	33525062	A	Intronic

SNP, single nucleotide polymorphism; Chr., Chromosome; Pos., position.

Ten independent SNPs showed association with both RA and r-axSpA (**Supplementary Table 2**). After Bonferroni correction for multiple testing (*p* < 3.33×10^−3^), the overlapping association signals remained statistically significant, supporting the robustness of the shared genetic architecture identified in the meta-analysis. As there was no significant heterogeneity between the UKBB and FinnGen datasets, these associations were further replicated in independent cohorts from the REPAIR consortium, including 2,044 RA cases, 367 r-axSpA cases, and 2,150 controls (**Table 1**).

Notably, the pleiotropy-guided screening approach enabled the selection of shared variants that were subsequently validated and replicated in this independent cohort, supporting their robustness and biological relevance. A detailed description of these populations has been previously reported (25, 31, 32). Briefly, the mean age of the RA and r-axSpA patients was 54.17 ± 13.77 and 46.05 ± 12.14, respectively, and the female-to-male ratio was 3.58 for RA (233/344) and 0.31 for r-axSpA (86/281; **Table 1**), consistent with their known sex bias.

The meta-analysis of the three large European populations (UKBB, FinnGen, and REPAIR) confirmed overlapping associations of *CARMIL1*, *GRM4*, *HTT*, *ITPR3*, *IKZF1*, *MANEA*, *MGAM2, PRSS16*, and *ZNF322* SNPs with the risk of developing RA and r-axSpA (**Table 3**). One SNP in *BTN3A2* (rs9393716) was excluded due to significant heterogeneity across studies (P_het_<0.05).

**Table 3:** Meta-analysis of the 11 SNPs associated with risk of developing IMID in three large European cohorts (UKBB, FinnGen and REPAIR).

RA discovery population												
SNP	Chr.	Nearest gene	Minor allele	UKBB N= 367,942 (4,380 cases/363,562 controls)		FinnGen N= 153,457 (6,236 cases/147,221 controls)		REPAIR N=4,079 (2,044 cases/2,035 controls)		Meta-analysis N=525,478 (12,660 cases/512,818 controls)		
				OR (95% CI)	*P*	OR (95% CI)	*P*	OR (95% CI)	*P*	OR (95% CI)	*P*	*P_Het_*
rs363075	4	*HTT*	A	1.12 (1.02-1.22)	**0.015**	1.13 (1.02-1.25)	**0.015**	0.99 (0.79-1.24)	0.917	**1.11 (1.04-1.18)**	**1.02×10^-3^**	0.561
rs1977199	6	*BTN2A1*	A	0.94 (0.90-0.99)	**0.026**	**0.92 (0.87-0.96)**	**3.74×10^-4^**	0.89 (0.77-1.03)	0.084	**0.93 (0.90-0.96)**	**1.26×10^-5^**	0.571
rs6901425	6	*ZNF322*	G	0.96 (0.90-1.02)	0.210	**0.88 (0.84-0.93)**	**3.00×10^-6^**	0.84 (0.67-1.05)	0.134	**0.91 (0.87-0.94)**	**2.04×10^-6^**	0.117
rs9393716	6	*BTN3A2*	G	**1.10 (1.04-1.16)**	**4.00×10^-4^**	**1.08 (1.03-1.14)**	**3.01×10^-3^**	0.93 (0.80-1.09)	0.380	**1.08 (1.04-1.12)**	**2.36×10^-5^**	0.107
rs12718261	7	*IKZF1*	A	1.06 (1.01-1.10)	**0.022**	**1.07 (1.02-1.10)**	**3.12×10^-3^**	**0.87 (0.77-0.99)**	**0.029**	**1.04 (1.02-1.08)**	**2.92×10^-3^**	0.004
rs66462181	6	*H2BC11*	C	1.11 (1.03-1.18)	**4.00×10^-3^**	1.12 (1.02-1.20)	**0.018**	**1.36 (1.07-1.74)**	**0.014**	**1.12 (1.06-1.18)**	**7.83×10^-5^**	0.484
rs72831267	6	*CARMIL1*	C	0.95 (0.91-0.99)	**0.020**	**0.93 (0.89-0.97)**	**7.99×10^-4^**	0.99 (0.87-1.13)	0.912	**0.94 (0.91-0.97)**	**6.36×10^-5^**	0.605
rs72843633	6	*PRSS16*	T	0.98 (0.91-1.05)	0.550	**0.89 (0.85-0.94)**	**3.40×10^-5^**	0.90 (0.71-1.12)	0.340	**0.92 (0.88-0.96)**	**1.16×10^-4^**	0.152
rs72920280	6	*MANEA*	T	**1.13 (1.06-1.21)**	**3.30×10^-4^**	1.08 (1.02-1.14)	**0.013**	0.92 (0.76-1.10)	0.349	**1.09 (1.04-1.13)**	**1.51×10^-4^**	0.072
rs73158426	7	*MGAM2*	G	1.14 (1.02-1.27)	**0.017**	1.22 (1.06-1.40)	**5.35×10^-3^**	1.06 (0.80-1.42)	0.679	**1.16 (1.07-1.26)**	**3.47×10^-4^**	0.639
rs77601296	6	*ITPR3*	A	0.93 (0.86-1-00)	**0.042**	**0.89 (0.84-0.94)**	**2.70×10^-5^**	0.85 (0.70-1.04)	0.123	**0.90 (0.86-0.94)**	**1.23×10^-6^**	0.502
rs2495964	6	*GRM4*	G	0.97 (0.92-1.01)	0.150	**0.90 (0.86-0.95)**	**5.23×10^-6^**	**0.86 (0.76-0.97)**	**0.016**	**0.93 (1.05-1.11)**	**4.58×10^-7^**	0.041
rs9469540	6	*ITPR3*	T	**0.91 (0.87-0.95)**	**3.70×10^-5^**	**0.91 (0.88-0.96)**	**5.82×10^-5^**	0.90 (0.80-1.02)	0.091	**0.91 (0.89-0.94)**	**1.52×10^-9^**	0.947
AS discovery population												
SNP	Chr.	Nearest gene	Minor allele	UKBB N=364,179 (617 cases/363,562 controls)		FinnGen N=166,144 (1,462 cases/164,682 controls)		REPAIR N=2,517 (367 cases/2,517 controls)		Meta-analysis N=532,840 (2,294 cases/528,459 controls)		
				OR (95% CI)	*P*	OR (95% CI)	*P*	OR (95% CI)	*P*	OR (95% CI)	*P*	*P_Het_*
rs363075	4	*HTT*	A	**1.21 (0.96-1.53)**	**6.30×10^-3^**	**1.39 (1.15-1.68)**	**5.76×10^-4^**	1.29 (0.89-1.86)	0.180	**1.31 (1.14-1.51)**	**1.33×10^-4^**	0.661
rs1977199	6	*BTN2A1*	A	**1.15 (1.01-1.32)**	**4.70×10^-3^**	**1.23 (1.12-1.35)**	**1.25×10^-5^**	**1.38 (1.16-1.64)**	**0.009**	**1.23 (1.15-1.32)**	**6.49×10^-9^**	0.268
rs6901425	6	*ZNF322*	G	0.91 (0.76-1.09)	0.100	**0.74 (0.67-0.82)**	**1.23×10^-8^**	1.17 (0.82-1.68)	0.377	**0.80 (0.73-0.87)**	**2.04×10^-7^**	0.027
rs9393716	6	*BTN3A2*	G	**0.87 (0.76-1.00)**	**0.033**	**0.87 (0.79-0.96)**	**4.88×10^-3^**	**0.61 (0.47-0.81)**	**4.50×10^-4^**	**0.82 (0.76-0.88)**	**2.84×10^-8^**	**0.001**
rs12718261	7	*IKZF1*	A	1.19 (1.05-1.34)	0.180	**1.14 (1.06-1.23)**	**5.16×10^-4^**	0.95 (0.77-1.10)	0.639	**1.13 (1.07-1.20)**	**4.84×10^-5^**	0.173
rs66462181	6	*H2BC11*	C	0.82 (0.68-0.98)	0.150	**0.78 (0.65-0.93)**	**5.29×10^-3^**	0.63 (0.38-1.05)	0.079	**0.77 (0.69-0.87)**	**1.72×10^-5^**	0.395
rs72831267	6	*CARMIL1*	C	**0.92 (0.82-1.04)**	**0.049**	**0.82 (0.75-0.89)**	**9.23×10^-7^**	0.99 (0.80-1.24)	0.964	**0.86 (0.81-0.92)**	**3.83×10^-6^**	0.108
rs72843633	6	*PRSS16*	T	**0.87 (0.72-1.06)**	**0.019**	**0.74 (0.67-0.82)**	**1.02×10^-8^**	1.30 (0.86-1.00)	0.208	**0.78 (0.71-0.85)**	**4.31×10^-8^**	0.054
rs72920280	6	*MANEA*	T	1.20 (1.01-1.43)	0.080	**1.17 (1.04-1.30)**	**6.59×10^-3^**	1.26 (0.93-1.69)	0.139	**1.18 (1.08-1.29)**	**3.69×10^-4^**	0.918
rs73158426	7	*MGAM2*	G	1.41 (1.07-1.85)	0.059	**1.44 (1.10-1.88)**	**7.22×10^-3^**	1.44 (0.91-2.27)	0.120	**1.42 (1.19-1.71)**	**1.58×10^-4^**	0.991
rs77601296	6	*ITPR3*	A	**0.84 (0.70-1.02)**	**0.013**	**0.76 (0.68-0.84)**	**5.50×10^-7^**	1.00 (0.72-1.40)	0.978	**0.79 (0.72-0.87)**	**5.27×10^-7^**	0.223
rs2495964	6	*GRM4*	G	**0.81 (0.66-0.95)**	**4.70×10^-3^**	**0.91 (0.82-0.99)**	**0.026**	0.92 (0.74-1.14)	0.443	**0.90 (1.05-1.19)**	**5.16×10^-4^**	0.501
rs9469540	6	*ITPR3*	T	**0.87 (0.78-0.97)**	**0.013**	**0.83 (0.76-0.90)**	**7.12×10^-6^**	1.07 (0.87-1-31)	0.532	**0.86 (0.81-0.92)**	**3.00×10^-6^**	0.102

SNP, single nucleotide polymorphism; OR, Odds Ratio; CI, Confidence Interval.

*P* values < 0.05 in bold. Bonferroni significant threshold was set up to *p* = 0.0033 (0.05/15 SNPs).

The *BTN3A2*_rs9393716_ SNP was excluded from the study due to significant heterogeneity in the meta-analysis.

**Figure 1:**
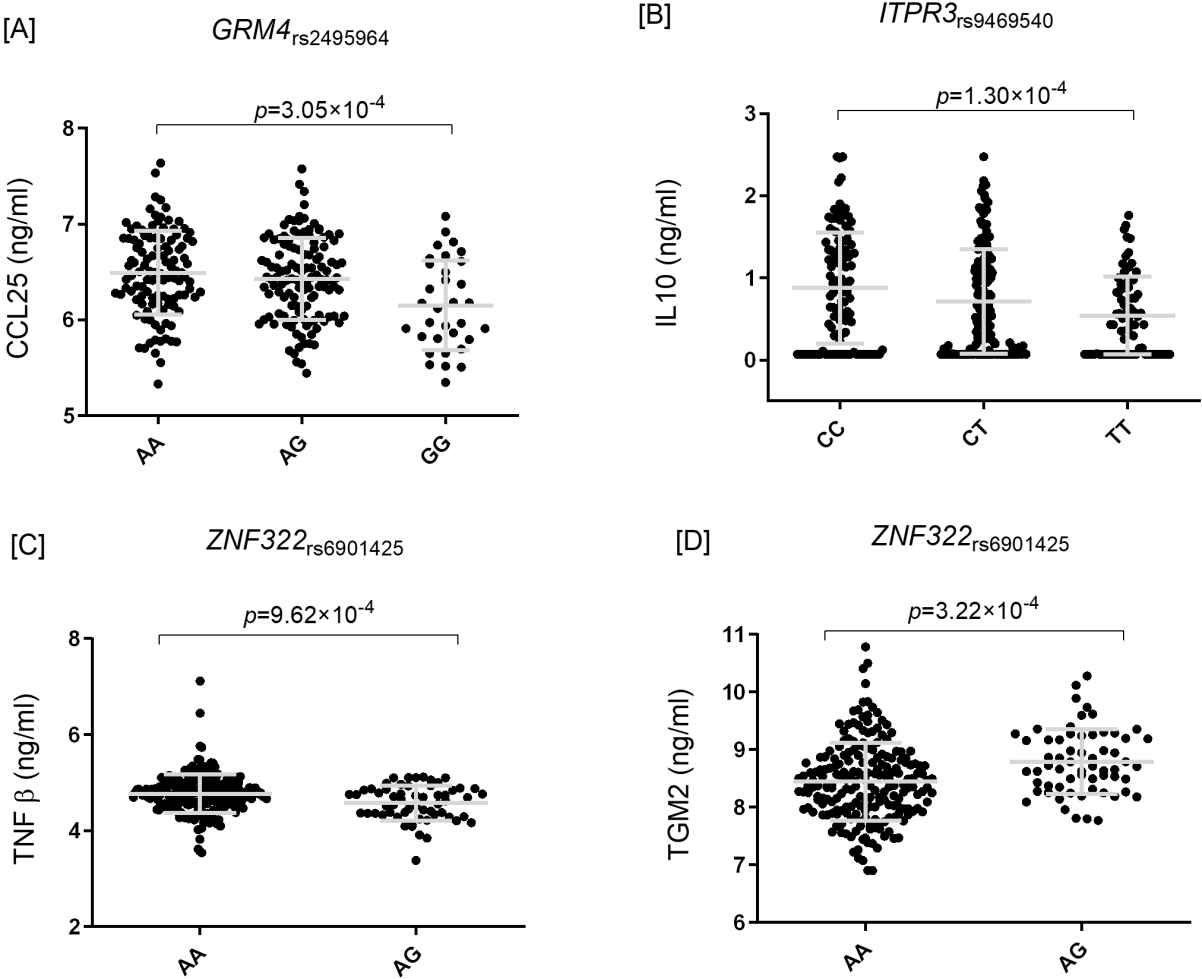
Functional characterization of identified genetic variants. Cytokine and protein concentrations were measured in peripheral blood mononuclear cells (PBMCs) or plasma samples from the 500 Functional Genomics (500FG; n=408 healthy controls, “HC”) and 300 Obese (300OB; n=302 overweight/obese individuals, “OB”) cohorts of the Human Functional Genomics Project (HFGP). PBMCs were stimulated *in vitro* with lipopolysaccharide (LPS; 1 ng/ml, 24 h) or *Staphylococcus aureus* (1×10^6^ CFU/ml, 7 days), and cytokine production was quantified using ELISA or multiplex immunoassays. **(A)** Decreased circulating CCL25 concentrations in obese carriers of the *GRM4*_rs2495964G_ protective allele. **(B)** Reduced IL10 production after LPS stimulation in carriers of the *ITPR3*_rs9469540T_ allele. **(C, D)** Decreased TNFβ and increased TGM2 concentrations in carriers of the *ZNF322*_rs6901425G_ allele. Error bars represent mean ± SD. Statistical significance was assessed using linear regression models adjusted for age and sex; p-values are indicated above each comparison. Abbreviations: HC, healthy controls; Ob, obese individuals; PBMC, peripheral blood mononuclear cell; LPS, lipopolysaccharide; RA, rheumatoid arthritis; r-axSpA, radiographic axial spondyloarthritis; SNP, single nucleotide polymorphism. The functional effects of the *ZNF322*_rs6901425_ SNP are based on its proxy variants rs72841519 and rs9467729 (D’ > 0.81, r² = 0.61).

Carriers of the *HTT*_rs363075A_, *IKZF1*_rs12718261A_, *MANEA*_rs72920280T_, and *MGAM2*_rs73158426G_ alleles had an increased risk of both RA and r-axSpA (OR_META-RA_=1.11/OR_META-AS_=1.31; OR_META-RA_=1.04/OR_META-AS_=1.13; OR_META-RA_=1.09/OR_META-AS_=1.18; and OR_META-RA_=1.16/OR_META-AS_=1.42), whereas carriers of the *CARMIL1*_rs72831267C_, *GRM4*_rs2495964T_, *ITPR3*_rs77601296A_, *ITPR3*_rs9469540T_, *PRSS16*_rs72843633T_, and *ZNF322*_rs6901425C_ alleles showed a decreased risk of both diseases (OR_META-RA_=0.94/OR_META-AS_=0.86; OR_META-RA_=0.93/OR_META-AS_=0.90; OR_META-RA_=0.90/OR_META-AS_=0.79; OR_META-RA_=0.91/OR_META-AS_=0.86; OR_META-RA_=0.92/OR_META-AS_=0.78; and OR_META-RA_=0.91/OR_META-AS_=0.80; **Table 3**). At the functional level, obese carriers of the *GRM4*_rs2495964G_ protective allele had significantly decreased circulating concentrations of CCL25 (*p* = 0.00030; **Figure 1A**), a chemokine involved in T- and B-cell migration and adipose-immune crosstalk. Additionally, we found a statistically significant correlation between the *ITPR3*_rs9469540T_ allele and decreased circulating concentrations of IL10 protein after *in vitro* stimulation of PBMCs with LPS (*p* = 1.30×10^-4^; **Figure 1B**).

The functional effects of *ZNF322*_rs6901425_ were inferred from its proxy variants rs72841519 and rs9467729 (D′>0.81, r²=0.61), as the lead SNP identified in the meta-analysis was not directly available in the functional genomics datasets used for the 500FG and 300OB cohorts. Therefore, proxy variants in strong LD were used to approximate the functional effects of the original locus. Obese carriers of the *ZNF322*rs6901425G protective allele showed significantly decreased circulating TNFβ (*p* = 9.62×10^-4^, **Figure 1C**) and increased TGM2 concentrations (*p* = 3.22×10^-4^, **Figure 1D**), both involved in immune regulation and tissue remodeling.

**Figure 2:**
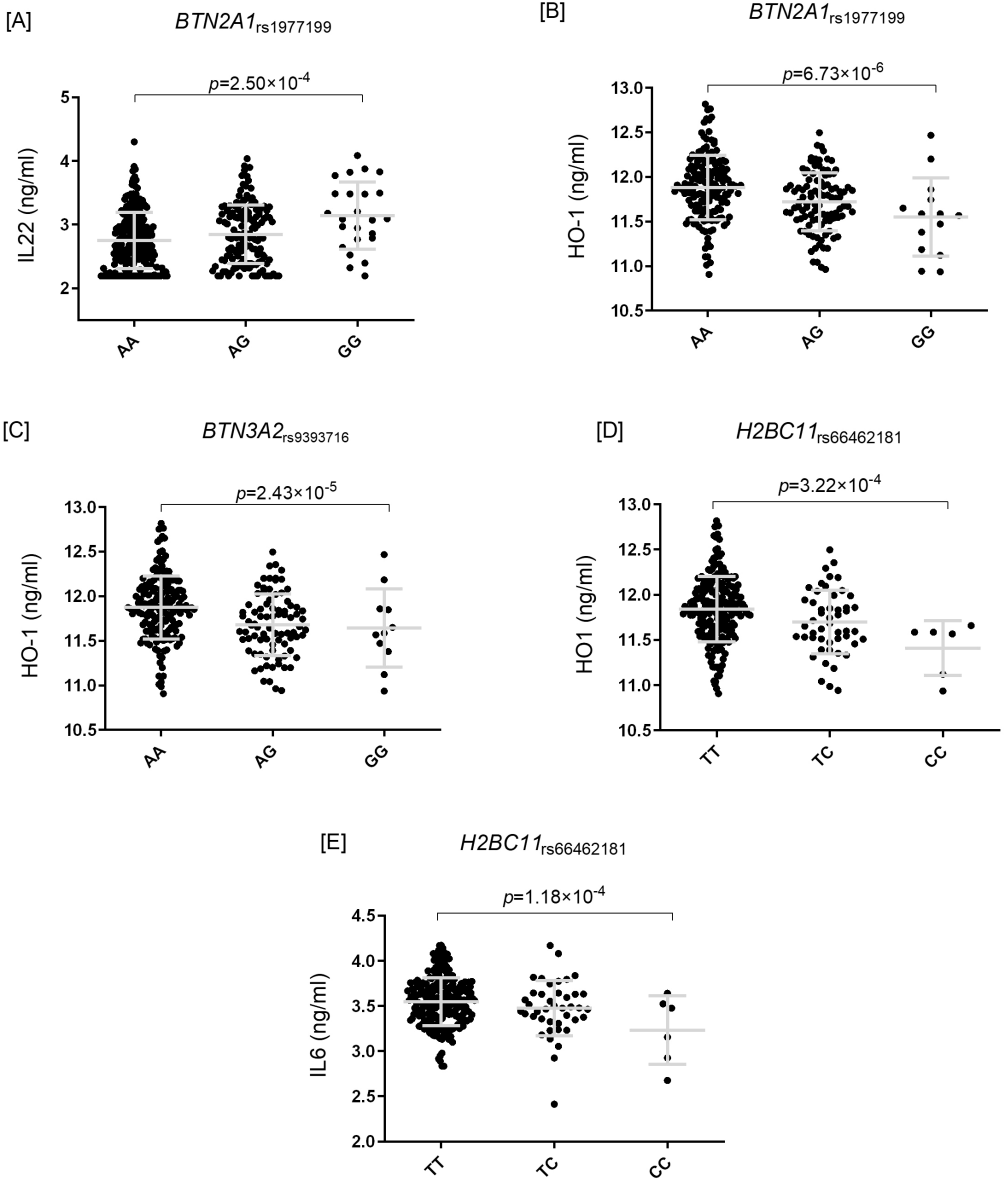
Functional characterization of identified genetic variants. Functional assays were performed in peripheral blood mononuclear cells (PBMCs) and plasma samples from two cohorts of the Human Functional Genomics Project (HFGP): the 500 Functional Genomics cohort (500FG; n=408 healthy controls, “HC”) and the 300 Obese cohort (300OB; n=302 overweight/obese individuals, “OB”). PBMCs were stimulated *in vitro* with *Staphylococcus aureus* (1×10^6^ CFU/ml, 7 days) or lipopolysaccharide (LPS; 1 ng/ml, 24 h), and cytokine production was measured by ELISA or multiplex immunoassay. **(A)** Decreased IL22 production after seven-day *S. aureus* stimulation in carriers of the *BTN2A1*_rs1977199A_ allele. **(B–D)** Altered circulating concentrations of heme oxygenase-1 (HO-1) in obese carriers of the *BTN2A1*_rs1977199A_, *BTN3A2*_rs9393716G_, and *H2BC11*_rs66462181C_ alleles, respectively. **(E)** Reduced IL6 secretion after *S. aureus* stimulation in PBMCs from *H2BC11*_rs66462181C_ carriers. Bars represent mean ± SD. Statistical significance was evaluated using linear regression adjusted for age and sex; p values are shown above each comparison. HC, healthy controls; Ob, obese individuals; PBMC, peripheral blood mononuclear cell; LPS, lipopolysaccharide; HO-1, heme oxygenase-1; RA, rheumatoid arthritis; r-axSpA, radiographic axial spondyloarthritis; SNP, single nucleotide polymorphism. Functional results of the *H2BC11*_rs66462181_ SNP were inferred from proxy variants rs13212562 and rs35909544 (D’>0.90 and r^2^ = 0.70).

Beyond the overlapping loci, several SNPs displayed opposite effects in RA and r-axSpA. Carriers of *BTN2A1*_rs1977199A_ had a decreased risk of RA (OR_META-RA_=0.93, *p* = 1.26×10^-5^) but an increased risk of r-axSpA (OR_META-AS_=1.23, *p* = 6.49×10^-9^). Conversely, carriers of *BTN3A2*_rs9393716G_ and *H2BC11*_rs66462181C_ alleles showed increased RA risk (OR_META-RA_=1.12, *p* = 7.83×10^-5^; and OR_META-RA_=1.08, *p* = 2.36×10^-5^), but decreased r-axSpA risk (OR_META-AS_=0.77, *p* = 1.72×10–*^5^* and OR_META-AS_=0.82, *p* = 2.84×10^-8^; **Table 3**). Functionally, *BTN2A1*_rs1977199A_ carriers produced less IL22 after *in vitro* stimulation of PBMCs with *Staphylococcus aureus* for seven days (*p* = 0.00025; **Figure 2A**), suggesting a differential contribution of IL22 to RA and r-axSpA pathogenesis.

In the 300OB cohort, obese carriers of *BTN2A1*_rs1977199A_, *BTN3A2*_rs9393716G_, and *H2BC11*_rs66462181C_ showed significantly altered circulating HO-1 concentrations (*p* = 6.73×10^-6^, *p* = 2.43×10–^5^ and *p* = 3.22×10^-4^; **Figures 2B–D**). *BTN2A1*_rs1977199A_ carriers had increased concentrations of HO-1, consistent with reduced RA risk but increased r-axSpA risk, whereas *BTN3A2*_rs9393716G_ and *H2BC11*_rs66462181C_ carriers showed the opposite trend. These findings suggest that variants at these loci may influence disease risk through modulation of HO-1 and its anti-inflammatory and cytoprotective effects.

PBMCs from *H2BC11*_rs66462181C_ carriers also produced significantly lower IL6 levels after *S. aureus* stimulation for 24h (*p* = 1.18×10^-4^; **Figure 2E**), suggesting a role of IL6 in the divergent immunological consequences of this locus in RA and r-axSpA. Finally, *BTN2A1*_rs1977199_ and *BTN3A2*_rs9393716_ (in moderate LD, r^2^ = 0.63) acted as eQTL for *BTN3A2, HMGN4*, *RP11-457M11.5*, and/or *TRIM38* across multiple tissues, whole blood, and lymphocytes (*p*-values ranging from *p* = 2.6×10–^6^ to 3.3×10^-36^).

We also evaluated whether the selected polymorphisms influenced the abundance of 91 blood-derived immune cell populations in the 500FG cohort. However, none of the analyzed SNPs showed statistically significant associations with immune cell subsets after correction for multiple testing. Similarly, no statistically significant associations were observed between the analyzed SNPs and circulating steroid hormone levels after correction for multiple testing (data not shown).

To ensure that these prioritized chromosome 6 signals were not driven by classical HLA effects, we evaluated linkage disequilibrium between the lead variants and established proxies of HLA risk alleles. All pairwise LD estimates showed r² < 0.01, indicating absence of meaningful LD with classical HLA risk variants. Pairwise LD estimates between prioritized SNPs and HLA proxies are shown in **Supplementary Table 1**.

**Figure 3:**
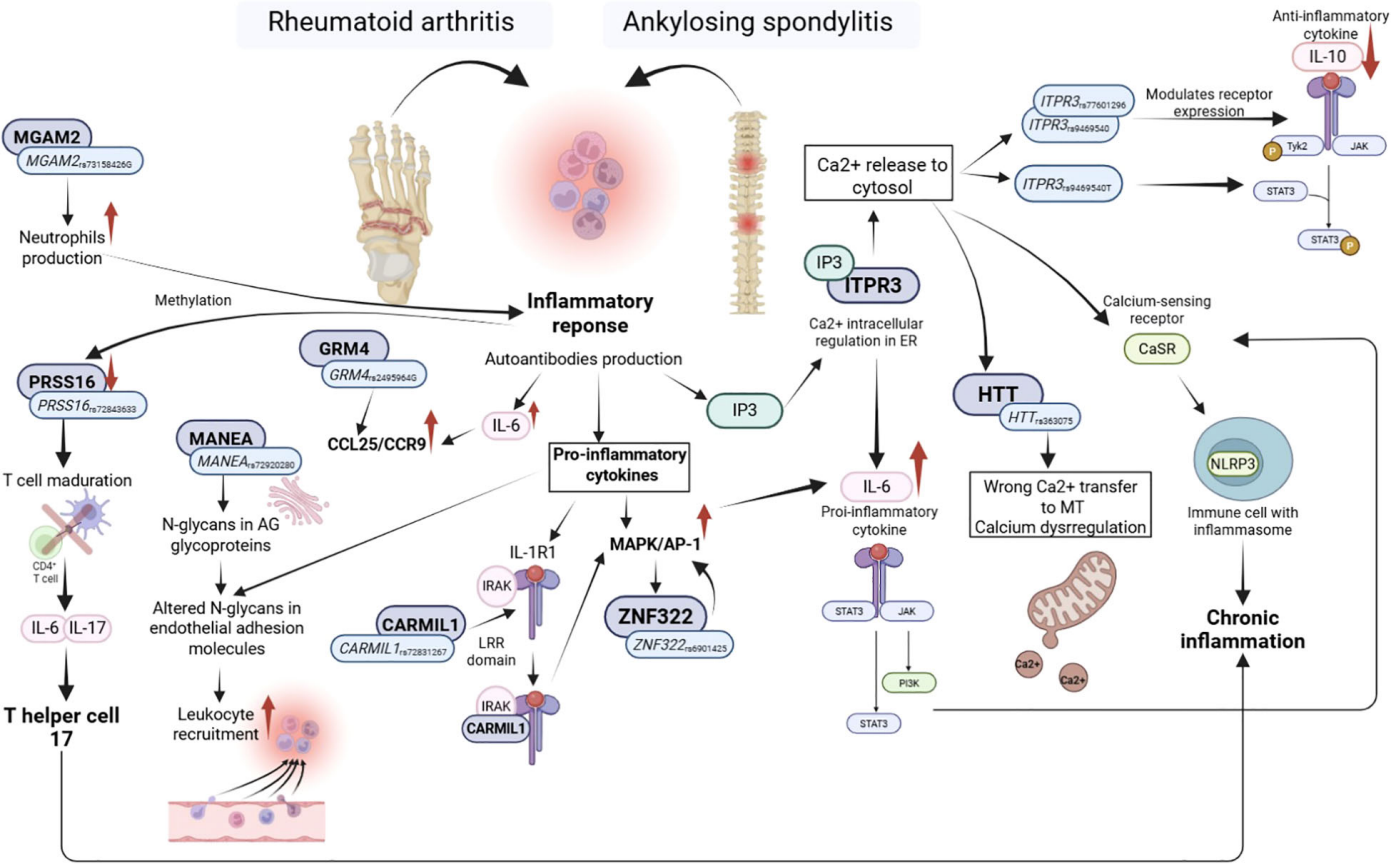
Proposed immunogenetic links and functional pathways underlying RA and AS risk.

In summary, the identified genetic variants support a coordinated regulation of immune pathways underlying susceptibility to RA and r-axSpA. Risk alleles in *HTT*, *IKZF1*, *MANEA*, and *MGAM2* were associated with increased disease risk, whereas protective alleles in *CARMIL1*, *GRM4*, *ITPR3*, *PRSS16*, and *ZNF322* were linked to reduced susceptibility, often through functional effects on cytokine levels and immune signaling. Variants in *GRM4* and *ITPR3* influenced CCL25 and IL10 concentrations, respectively, while *ZNF322* was associated with changes in TNFβ and TGM2 concentrations. *BTN2A1*, *BTN3A2*, and *H2BC11* variants showed opposing effects on RA and r-axSpA risk, likely through modulation of HO-1 and IL6. Together, these observations point to shared yet distinct immunogenetic mechanisms driving RA and r-axSpA (**Figure 3**).

## Discussion

This study provides the first large-scale evidence of shared and disease-specific genetic architecture between RA and/or r-axSpA, identifying novel loci and functional pathways that may explain their overlapping but distinct immunopathology. Given the strong immunogenetic contribution of the MHC region to both RA and r-axSpA, our integrative framework was designed to capture immune-related shared architecture rather than to focus exclusively on non-MHC locus discovery. This approach allows a more biologically informed interpretation of overlapping risk patterns across immune-mediated diseases. This strategy aligns with pleiotropy-informed GWAS frameworks that leverage cross-disease genetic overlap to improve discovery power and characterize shared pathogenic mechanisms. Through a meta-analysis of three large European populations and integration with functional genomics datasets, we uncovered 10 genetic variants shared by both diseases and additional loci with opposite effects, revealing complex patterns of immune regulation.

### Shared genetic background between RA and r-axSpa

Several loci including *CARMIL1*, *GRM4*, *HTT*, *ITPR3*, *IKZF1*, *MANEA*, *MGAM2, PRSS16*, and *ZNF322* show a consistent association with susceptibility to both RA and r-axSpA. Importantly, several prioritized overlapping loci were located outside the classical HLA region and were not in strong LD with previously reported GWAS signals, supporting the identification of novel susceptibility signals beyond established loci. These genes converge on pathways related to T-cell activation, intracellular signaling, and regulation of inflammatory cytokines, supporting the concept of a common immunogenetic core among IMIDs.

Among these, *ITPR3* and *ZNF322* emerged as the most significant overlapping loci. *ITPR3*, located on chromosome 6, encodes Inositol 1,4,5-trisphosphate receptor type 3, an intracellular Ca²^+^ channel involved in T-cell receptor signaling and apoptosis regulation. Previous GWAS have linked *ITPR3* variants to autoimmune diseases, including RA (33), type 1 diabetes (T1D) (34), systemic lupus erythematosus (SLE) (35), and Graves’ disease (36), highlighting its broad immunoregulatory role. The *ITPR3*_rs77601296_ and *ITPR3*_rs9469540_ SNPs identified here are independent of previously reported GWAS hits (rs2229634, rs999943, rs3748079, rs210122) (33, 35), suggesting a more complex genetic structure. Protective alleles likely modulate receptor function to prevent excessive immune activation, consistent with reduced IL10 secretion following LPS stimulation and eQTL effects on *ITPR3* and *BAK1* across multiple cell types. Moreover, these alleles affect chromatin states and alter histone modifications in key immune cell types, including monocytes, T regulatory cells, CD8^+^ T cells, B cells, NK cells, and neutrophils.

*ZNF322*, located on chromosome 6p22.3, encodes a zinc-finger transcription factor (37) that regulates cell proliferation and differentiation via MAPK (mitogen-activated protein kinase) signaling (38), a pathway central to inflammatory responses and joint damage in RA. Although *ZNF322* has been previously linked to RA via transcriptome-wide association studies (TWAS) showing overexpression in RA patients compared with controls (39, 40), our study is the first to implicate this gene in r-axSpA. Functionally, carriers of the *ZNF322*_rs6901425G_ protective allele exhibited reduced TNFβ and increased TGM2 concentrations, pointing to immunomodulatory and tissue-remodeling mechanisms shared across both diseases.

Other shared loci highlight convergent biological processes. *HTT* and *CARMIL1* are involved in cytoskeletal remodeling and fibroblast invasiveness, key processes in synovial hyperplasia and joint destruction. *HTT* encodes the huntingtin protein, essential for intracellular transport, and neuronal survival, and although not previously linked to r-axSpA, prior studies have reported its role in RA pathogenesis through fibroblast-like synoviocytes (FLS) (41), which are central to joint damage. The HTT-interacting protein HIP1 promotes FLS invasiveness via Rac1 signaling and other cellular processes like cytoskeletal organization and receptor endocytosis (42). Studies have shown that HIP1 deficiency reduces FLS invasion by about 50%, highlighting its potential role in RA susceptibility and severity. Elevated HIP1 autoantibodies also correlate with disease progression (43). In silico analyses indicate that *HTT*_rs363075_ modulates osteoblast activity through H3K4me1 and H3K27ac histone modifications (44).

*CARMIL1* (LRRC16A) encodes a protein regulating actin dynamics and IL1 signaling, influencing fibroblast migration and extracellular matrix remodeling (45–47). Although this is the first report linking *CARMIL1* directly to RA and r-axSpA susceptibility, multi-trait GWAS have identified this locus in RA, T1D, and Graves’ disease (34).

Similarly, *GRM4*, encoding a metabotropic glutamate receptor, modulates intracellular cAMP signaling and immune activation. Carriers of the protective *GRM4*_rs2495964G_ allele had lower circulating CCL25 levels, a chemokine that binds CCR9 (48, 49) and it overexpressed in both RA and r-axSpA (50). The CCL25/CCR9 axis promotes monocyte migration, polarization, and osteoclastogenesis (50, 51) and its blockade reduces arthritis severity in experimental models (52). These findings implicate *GRM4* in CCL25-mediated immune cell trafficking and inflammation.

Other loci such as *IKZF1*, *MANEA*, *MGAM2*, and *PRSS16* may contribute to immune regulation. *IKZF1* has been linked to RA in Han Chinese populations (53), consistent with its role in T-cell differentiation and DNA methylation control. *MANEA* and *MGAM2*, involved in glycan trimming (54) and immune-related gene expression (55), respectively, have not been directly associated with autoimmune diseases, thought their functions suggest plausible roles in immune modulation. A Mendelian randomization study found no evidence supporting the involvement of *MANEA* or *MGAM2* in r-axSpA (56). *PRSS16*, a thymic serine protease critical for T-cell selection and tolerance (57), is differentially methylated in RA (58) and was associated with seropositive RA in multi-ancestry GWAS (4). Although not in LD with *PRSS16*_rs72843633_ in Europeans (r (2) = 0.0019), these findings collectively reinforce *PRSS16* as a shared susceptibility locus. Supporting the biological plausibility of these associations, in silico analyses revealed enrichment of active histone marks (H3K9ac) and modified binding motifs for transcription factors including AP-1 (activator protein 1), p300, HDAC2 (histone deacetylase 2), Foxp3 (forkhead box P3), and Zbtb3 (zinc finger and BTB domain-containing protein 3) (44), all implicated in T-cell differentiation and autoimmune regulation.

### Opposite genetic effects and disease divergence

For the first time, we identified inverse associations for variants in *BTN2A1*, *BTN3A2*, and *H2BC11*, which showed differential effects on RA and r-axSpA. The *BTN2A1*_rs1977199_ allele decreased RA risk but increased r-axSpA risk, while variants in *BTN3A2* and *H2BC11* exhibited the opposite pattern. Functional assays revealed that *BTN2A1*_rs1977199A_ carriers produced less IL22 after *Staphylococcus aureus* stimulation, consistent with differential IL22 contributions to RA and r-axSpA pathogenesis. IL22 is elevated in both RA and r-axSpA (59–61), but correlates with disease activity and bone erosions in RA. In RA, IL22 is mainly produced by Th17 and Th22 cells (60), as well as NK-22 cells and contributes to joint inflammation by stimulating FLS that produce MCP-1 (62). Importantly, blocking IL22 or its receptor has shown potential in experimental models to reduce inflammation and bone erosion, highlighting IL22 as a possible therapeutic target (60). In AS, IL22 is mainly produced by NKp44+ natural killer cells (63), suggesting a role in mucosal immunity and systemic inflammation via the IL23/IL22 axis. Additionally, IL22 has been shown to promote osteogenic differentiation of mesenchymal stem cells, potentially contributing to the abnormal bone formation characteristic of AS (64).

In the 300OB (300 Obesity) cohort, *BTN2A1*_rs1977199A_ carriers exhibited increased HO-1 (heme oxygenase-1) concentrations, aligning with reduced RA risk and higher r-axSpA risk, whereas *BTN3A2*_rs9393716G_ and *H2BC11*_rs66462181C_ carriers had lower HO-1, consistent with the opposite direction of risk. HO-1 is an anti-inflammatory enzyme that modulates oxidative stress, cytokine release, and the regulatory T cells/Th17 helper cells balance. Its expression attenuates inflammation in both RA and AS, though through disease-specific pathways. Elevated HO-1 correlates with lower TNF-α (tumor necrosis factor-alpha), IL6, and IL8 levels and with reduced oxidative stress and COX-2 (cyclooxygenase-2) expression in RA, while in AS it associates with bone metabolism markers such as BMP-7 (bone morphogenetic protein 7) and Runx2 (runt-related transcription factor 2). Pharmacological and natural HO-1 inducers, such as auranofin, quercetin, and resveratrol, have shown anti-inflammatory efficacy in both conditions (65). Conversely, HO-1 inhibition exacerbates inflammation (66). Thus, genetic modulation of HO-1 expression may explain opposite disease effects.

Supporting this interpretation, *H2BC11*_rs66462181C_ carriers exhibited reduced IL6 production after *Staphylococcus aureus* stimulation, implicating IL6 divergent immune outcomes. According to GTex data, these SNPs also acted as eQTL for *BTN3A2*, *HMGN4*, *RP11-457M11.5*, and *TRIM38* across blood and lymphocytes tissues, reinforcing their regulatory relevance.

### Strengths and limitations

The major strengths of this study include its large sample size, combining three independent European cohorts (15,106 IMID patients and ~530,000 controls), and the integration of genetic, epigenetic, and immunological datasets. Functional analyses in the 500FG and 300OB HFGP cohorts provided mechanistic support for how the identified variants modulate immune pathways.

One limitation of this study is that the analyses were restricted to individuals of European ancestry, which may limit the generalizability of our findings to other populations. Genetic susceptibility to r-axSpA and other immune-mediated inflammatory diseases can vary across ancestries, particularly within the HLA region where allele frequencies and subtype distributions (e.g., HLA-B27 variants) differ substantially between populations. Therefore, further multi-ancestry studies will be necessary to determine the transferability of the loci identified here.

Another limitation is the imbalance in the number of cases between RA and r-axSpA in the discovery phase. Although this difference reflects the lower prevalence of r-axSpA in population-based cohorts such as UK Biobank and FinnGen, the smaller number of r-axSpA cases may reduce statistical power to detect variants with modest effects that are specific to this condition.

A further limitation concerns the geographic representation of r-axSpA cases in the REPAIR validation cohort. While RA samples were available from several participating countries, r-axSpA cases were only available from Spain and Poland, reflecting the availability of samples within the consortium rather than an analytical selection. This difference is consistent with the lower prevalence of r-axSpA and the more limited recruitment of these patients across participating centers.

Another limitation is that functional analyses for some loci (e.g., *ZNF322* and *H2BC11*) were performed using proxy variants in strong linkage disequilibrium with the lead SNPs identified in the meta-analysis, because the original variants were not directly available in the functional genomics datasets used for the 500FG and 300OB cohorts. Although the use of proxy variants is a commonly adopted strategy in functional genomics studies, these results should be interpreted with caution because the observed functional effects may not fully reflect those of the original lead variants.

Finally, our analysis may have missed regulatory elements located beyond ±5 kb from the studied SNPs. Further fine-mapping and multi-ancestry studies are therefore warranted. Although the use of a pleiotropy-guided screening threshold may appear less stringent than conventional GWAS thresholds, this strategy enhances sensitivity to detect shared genetic architecture and was followed by independent replication and multiple-testing correction to ensure robustness.

## Conclusion

In summary, this study identifies *CARMIL1*, *GRM4*, *HTT*, *IKZF1*, *ITPR3*, *MANEA*, *MGAM2*, *PRSS16*, and *ZNF322* as shared susceptibility loci for RA and r-axSpA, and *BTN2A1*, *BTN3A2*, and *H2BC11* as disease-divergent loci. The shared variants modulate cytokine pathways such as IL10 and CCL25, whereas the opposing variants influence IL22, IL6, and HO-1 signaling. Together, these findings support a model of overlapping yet distinct immunogenetic mechanisms underpinning RA and r-axSpA, offering potential targets for cross-disease therapeutic strategies.

## Data Availability

The original contributions presented in the study are included in the article/**Supplementary Material**. Further inquiries can be directed to the corresponding author/s.

## References

[1] Alamanos Y Drosos A . Epidemiology of adult rheumatoid arthritis. Autoimmun Rev. (2005) 4:130–6. doi: 10.1016/j.autrev.2004.09.002. PMID: 15823498

[2] Burton PR Clayton DG Cardon LR Craddock N Deloukas P Duncanson A . Genome-wide association study of 14,000 cases of seven common diseases and 3,000 shared controls. Nature. (2007) 447:661–78. doi: 10.1038/nature05911. PMID: 17554300 PMC2719288

[3] Okada Y Terao C Ikari K Kochi Y Ohmura K Suzuki A . Meta-analysis identifies nine new loci associated with rheumatoid arthritis in the Japanese population. Nat Genet. (2012) 44:511–6. doi: 10.1038/ng.2231. PMID: 22446963

[4] Ishigaki K Sakaue S Terao C Luo Y Sonehara K Yamaguchi K . Multi-ancestry genome-wide association analyses identify novel genetic mechanisms in rheumatoid arthritis. Nat Genet. (2022) 54:1640–51. doi: 10.1038/s41588-022-01213-w. PMID: 36333501 PMC10165422

[5] van der Helm-van Mil AHM Wesoly JZ Huizinga TWJ . Understanding the genetic contribution to rheumatoid arthritis. Curr Opin Rheumatol. (2005) 17:299–304. doi: 10.1097/01.bor.0000160780.13012.be. PMID: 15838240

[6] Saevarsdottir S Stefansdottir L Sulem P Thorleifsson G Ferkingstad E Rutsdottir G . Multiomics analysis of rheumatoid arthritis yields sequence variants that have large effects on risk of the seropositive subset. Ann Rheum Dis. (2022) 81:1085–95. doi: 10.1136/annrheumdis-2021-221754. PMID: 35470158 PMC9279832

[7] Verma A Huffman JE Rodriguez A Conery M Liu M Ho Y-L . Diversity and scale: Genetic architecture of 2068 traits in the VA Million Veteran Program. Science. (2024) 385:eadj1182. doi: 10.1126/science.adj1182. PMID: 39024449 PMC12857194

[8] van der Linden MPM Feitsma AL le Cessie S Kern M Olsson LM Raychaudhuri S . Association of a single-nucleotide polymorphism in CD40 with the rate of joint destruction in rheumatoid arthritis. Arthritis Rheum. (2009) 60:2242–7. doi: 10.1002/art.24721. PMID: 19644859 PMC3121053

[9] Liang YL Wu H Shen X Li P-Q Yang X-Q Liang L . Association of STAT4 rs7574865 polymorphism with autoimmune diseases: a meta-analysis. Mol Biol Rep. (2012) 39:8873–82. doi: 10.1007/s11033-012-1754-1. PMID: 22714917

[10] Parkes M Cortes A van Heel DA Brown MA . Genetic insights into common pathways and complex relationships among immune-mediated diseases. Nat Rev Genet. (2013) 14:661–73. doi: 10.1038/nrg3502. PMID: 23917628

[11] Eyre S Hinks A Bowes J Flynn E Martin P Wilson AG . Overlapping genetic susceptibility variants between three autoimmune disorders: rheumatoid arthritis, type 1 diabetes and coeliac disease. Arthritis Res Ther. (2010) 12:R175. doi: 10.1186/ar3139. PMID: 20854658 PMC2991006

[12] Cotsapas C Voight BF Rossin E Lage K Neale BM Wallace C . Pervasive sharing of genetic effects in autoimmune disease. PloS Genet. (2011) 7:e1002254. doi: 10.1371/journal.pgen.1002254. PMID: 21852963 PMC3154137

[13] Ellinghaus D Jostins L Spain SL Cortes A Bethune J Han B . Analysis of five chronic inflammatory diseases identifies 27 new associations and highlights disease-specific patterns at shared loci. Nat Genet. (2016) 48:510–8. doi: 10.1038/ng.3528. PMID: 26974007 PMC4848113

[14] Saurabh R Fouodo CJK König IR Busch H Wohlers I . A survey of genome-wide association studies, polygenic scores and UK Biobank highlights resources for autoimmune disease genetics. Front Immunol. (2022) 13:972107. doi: 10.3389/fimmu.2022.972107. PMID: 35990650 PMC9388859

[15] Flores-Robles BJ Labrador-Sánchez E Andrés-Trasahedo E Pinillos-Aransay V Joven-Zapata M-Y Torrecilla Lerena L . Concurrence of rheumatoid arthritis and ankylosing spondylitis: Analysis of seven cases and literature review. Case Rep Rheumatol. (2022) 2022:1–7. doi: 10.1155/2022/8500567. PMID: 35669458 PMC9167098

[16] Smith JA . Update on ankylosing spondylitis: current concepts in pathogenesis. Curr Allergy Asthma Rep. (2015) 15:489. doi: 10.1007/s11882-014-0489-6. PMID: 25447326

[17] Kim HJ Nemani VM Riew KD Brasington R . Cervical spine disease in rheumatoid arthritis: incidence, manifestations, and therapy. Curr Rheumatol Rep. (2015) 17:9. doi: 10.1007/s11926-014-0486-8. PMID: 25663179

[18] Chudasama YV Khunti K Coles B Gillies CL Islam N Rowlands AV . Life expectancy following a cardiovascular event in individuals with and without type 2 diabetes: A UK multi-ethnic population-based observational study. Nutr Metab Cardiovasc Dis. (2023) 33:1358–66. doi: 10.1016/j.numecd.2023.04.003. PMID: 37169664

[19] Kisacik B Tufan A Kalyoncu U Karadag O Akdogan A Ozturk MA . Mean platelet volume (MPV) as an inflammatory marker in ankylosing spondylitis and rheumatoid arthritis. Joint Bone Spine. (2008) 75:291–4. doi: 10.1016/j.jbspin.2007.06.016. PMID: 18403245

[20] Macfarlane GJ MacDonald RIR Pathan E Siebert S Gaffney K Choy E . Influence of co-morbid fibromyalgia on disease activity measures and response to tumour necrosis factor inhibitors in axial spondyloarthritis: results from a UK national register. Rheumatol (Oxford). (2018) 57:1982–90. doi: 10.1093/rheumatology/key206. PMID: 30053166 PMC6199528

[21] Gagliano Taliun SA VandeHaar P Boughton AP Welch RP Taliun D Schmidt EM . Exploring and visualizing large-scale genetic associations by using PheWeb. Nat Genet. (2020) 52:550–2. doi: 10.1038/s41588-020-0622-5. PMID: 32504056 PMC7754083

[22] Bycroft C Freeman C Petkova D Band G Elliott LT Sharp K . The UK Biobank resource with deep phenotyping and genomic data. Nature. (2018) 562:203–9. doi: 10.1038/s41586-018-0579-z. PMID: 30305743 PMC6786975

[23] Peterson RE Kuchenbaecker K Walters RK Chen C-Y Popejoy AB Periyasamy S . Genome-wide Association Studies in Ancestrally Diverse Populations: Opportunities, Methods, Pitfalls, and Recommendations. Cell. (2019) 179:589–603. doi: 10.1016/j.cell.2019.08.051. PMID: 31607513 PMC6939869

[24] Willer CJ Li Y Abecasis GR . METAL: fast and efficient meta-analysis of genomewide association scans. Bioinformatics. (2010) 26:2190–1. doi: 10.1093/bioinformatics/btq340. PMID: 20616382 PMC2922887

[25] Sánchez-Maldonado JM Cáliz R López-Nevot MÁ Cabrera-Serrano AJ Moñiz-Díez A Canhão H . Validation of GWAS-identified variants for anti-TNF drug response in rheumatoid arthritis: A meta-analysis of two large cohorts. Front Immunol. (2021) 12:672255. doi: 10.3389/fimmu.2021.672255. PMID: 34777329 PMC8579100

[26] Aletaha D Neogi T Silman AJ Funovits J Felson DT Bingham 3rd CO . Rheumatoid arthritis classification criteria: An American College of Rheumatology/European League Against Rheumatism collaborative initiative. Arthritis Rheum. (2010) 62:2569–81. doi: 10.1136/ard.2010.138461. PMID: 20872595

[27] Clavero E Sanchez-Maldonado JM Macauda A Ter Horst R Sampaio-Marques B Jurczyszyn A . Polymorphisms within autophagy-related genes as susceptibility biomarkers for multiple myeloma: A meta-analysis of three large cohorts and functional characterization. Int J Mol Sci. (2023) 24(10):8500. doi: 10.3390/ijms24108500. PMID: 37239846 PMC10218542

[28] Li Y Oosting M Smeekens SP Jaeger M Aguirre-Gamboa R Le KTT . A functional genomics approach to understand variation in cytokine production in humans. Cell. (2016) 167:1099–1110.e14. doi: 10.1016/j.cell.2016.10.017. PMID: 27814507

[29] Ríos-Tamayo R Lupiañez CB Campa D Hielscher T Weinhold N Martínez-López J . A common variant within the HNF1B gene is associated with overall survival of multiple myeloma patients: results from the IMMEnSE consortium and meta-analysis. Oncotarget. (2016) 7:59029–48. doi: 10.18632/oncotarget.10665. PMID: 27437873 PMC5312293

[30] García-Martín P Díez AM Maldonado JMS Moñiz Díez A Maldonado JSS Cabrera Serrano AJ . Validation and functional characterization of GWAS-identified variants for chronic lymphocytic leukemia: a CRuCIAL study. Blood Cancer J. (2022) 12:79. doi: 10.1038/s41408-022-00676-8. PMID: 35581176 PMC9114372

[31] Canet LM Filipescu I Cáliz R Lupiañez CB Canhão H Escudero A . Genetic variants within the TNFRSF1B gene and susceptibility to rheumatoid arthritis and response to anti-TNF drugs. Pharmacogenet Genomics. (2015) 25:323–33. doi: 10.1097/fpc.0000000000000140. PMID: 25850964

[32] Sánchez-Maldonado JM Martínez-Bueno M Canhão H Ter Horst R Muñoz-Peña S Moñiz-Díez A . NFKB2 polymorphisms associate with the risk of developing rheumatoid arthritis and response to TNF inhibitors: Results from the REPAIR consortium. Sci Rep. (2020) 10:4316. doi: 10.1038/s41598-020-61331-5. PMID: 32152480 PMC7062729

[33] Mo XB Sun YH Zhang YH Lei SF . Integrative analysis highlighted susceptibility genes for rheumatoid arthritis. Int Immunopharmacol. (2020) 86:106716. doi: 10.1016/j.intimp.2020.106716. PMID: 32599322

[34] Wen YP Yu ZG . Identifying shared genetic loci and common risk genes of rheumatoid arthritis associated with three autoimmune diseases based on large-scale cross-trait genome-wide association studies. Front Immunol. (2023) 14:1160397. doi: 10.3389/fimmu.2023.1160397. PMID: 37377963 PMC10291128

[35] Oishi T Iida A Otsubo S Kamatani Y Usami M Takeiet T . A functional SNP in the NKX2.5-binding site of ITPR3 promoter is associated with susceptibility to systemic lupus erythematosus in Japanese population. J Hum Genet. (2008) 53:151–62. doi: 10.1007/s10038-007-0233-3. PMID: 18219441

[36] Nakabayashi K Tajima A Yamamoto K Takahashi A Hata K Takashima Y . Identification of independent risk loci for Graves’ disease within the MHC in the Japanese population. J Hum Genet. (2011) 56:772–8. doi: 10.1038/jhg.2011.99. PMID: 21900946

[37] Li Y Wang Y Zhang C Yuan W Wang J Zhuet C . ZNF322, a novel human C2H2 Krüppel-like zinc-finger protein, regulates transcriptional activation in MAPK signaling pathways. Biochem Biophys Res Commun. (2004) 325:1383–92. doi: 10.1016/j.bbrc.2004.10.183. PMID: 15555580

[38] He S Sharpless NE . Senescence in health and disease. Cell. (2017) 169:1000–11. doi: 10.1016/j.cell.2017.05.015. PMID: 28575665 PMC5643029

[39] Ni J Wang P Yin KJ Yang X-K Cen H Suiet C . Novel insight into the aetiology of rheumatoid arthritis gained by a cross-tissue transcriptome-wide association study. RMD Open. (2022) 8:e002529. doi: 10.1136/rmdopen-2022-002529. PMID: 37582060 PMC9462377

[40] Ni J Wang P Yin KJ Yang X-K Cen H Sui C . Novel insight into the etiology of rheumatoid arthritis gained by a cross-tissue transcriptome-wide association study. SSRN Electron J. (2022). doi: 10.2139/ssrn.4117423. PMID: 37582060 PMC9462377

[41] Ai R Laragione T Hammaker D Boyle DL Wildberg A Maeshima K . Comprehensive epigenetic landscape of rheumatoid arthritis fibroblast-like synoviocytes. Nat Commun. (2018) 9:1921. doi: 10.1038/s41467-018-04310-9. PMID: 29765031 PMC5953939

[42] Laragione T Harris C Gulko PS . KIF1C and new Huntingtin-interacting protein 1 binding proteins regulate rheumatoid arthritis fibroblast-like synoviocytes’ phenotypes. Front Immunol. (2024) 15:1323410. doi: 10.3389/fimmu.2024.1323410. PMID: 38726004 PMC11079228

[43] Surbhi GA Chaturvedi V Verma S Rawat S Ganfuly NK Shivani AM . Huntingtin Interacting Protein 1 (HIP1) autoantibodies as a novel potential surrogate marker for Rheumatoid Arthritis: Pilot Study. bioRxiv. (2022). doi: 10.1101/2022.09.07.22279672. PMID: 41887800

[44] Ward LD Kellis M . HaploReg: a resource for exploring chromatin states, conservation, and regulatory motif alterations within sets of genetically linked variants. Nucleic Acids Res. (2012) 40:D930–4. doi: 10.1093/nar/gkr917. PMID: 22064851 PMC3245002

[45] Wang Q Notay K Downey GP McCulloch CA . The leucine-rich repeat region of CARMIL1 regulates IL-1-mediated ERK activation, MMP expression, and collagen degradation. Cell Rep. (2020) 31:107781. doi: 10.1016/j.celrep.2020.107781. PMID: 32610117 PMC8713033

[46] Stark BC Lanier MH Cooper JA . CARMIL family proteins as multidomain regulators of actin-based motility. Mol Biol Cell. (2017) 28:1713–23. doi: 10.1091/mbc.e17-01-0019. PMID: 28663287 PMC5491179

[47] Liang Y Niederstrasser H Edwards M Jackson CE Cooper JA . Distinct roles for CARMIL isoforms in cell migration. Mol Biol Cell. (2009) 20:5290–305. doi: 10.1091/mbc.e08-10-1071. PMID: 19846667 PMC2793302

[48] Papadakis KA Prehn J Nelson V Cheng L Binder SW Ponathet PD . The role of thymus-expressed chemokine and its receptor CCR9 on lymphocytes in the regional specialization of the mucosal immune system. J Immunol. (2000) 165:5069–76. doi: 10.4049/jimmunol.165.9.5069. PMID: 11046037

[49] Kunkel EJ Campbell DJ Butcher EC . Chemokines in lymphocyte trafficking and intestinal immunity. Microcirculation. (2003) 10:313–23. doi: 10.1080/713773645. PMID: 12851648

[50] Umar S Palasiewicz K Van Raemdonck K Volin MV Romay B Ahmadet I . CCL25 and CCR9 is a unique pathway that potentiates pannus formation by remodeling RA macrophages into mature osteoclasts. Eur J Immunol. (2021) 51:903–14. doi: 10.1002/eji.202048681. PMID: 33347617 PMC10041658

[51] Schmutz C Cartwright A Williams H Haworth O Williams JHH Fileret A . Monocytes/macrophages express chemokine receptor CCR9 in rheumatoid arthritis and CCL25 stimulates their differentiation. Arthritis Res Ther. (2010) 12:R161. doi: 10.1186/ar3120. PMID: 20738854 PMC2945064

[52] Yokoyama W Kohsaka H Kaneko K Walters M Takayasu A Fukuda S . Abrogation of CC chemokine receptor 9 ameliorates collagen-induced arthritis of mice. Arthritis Res Ther. (2014) 16:445. doi: 10.1186/s13075-014-0445-9. PMID: 25248373 PMC4201712

[53] Li D Sui X Hou Q Feng X Chen Y Chen X . Association between IKZF1 related gene polymorphism, DNA methylation and rheumatoid arthritis in Han Chinese: A case-control study. (2020). doi: 10.22541/au.159103361.19001792

[54] Sobala ŁF Fernandes PZ Hakki Z Davies GJ . Structure of human endo-α-1,2-mannosidase (MANEA), an antiviral host-glycosylation target. Proc Natl Acad Sci USA. (2020) 117:29595–601. doi: 10.1073/pnas.2013620117. PMID: 33154157 PMC7703563

[55] Xu S Feng Y Zhao S . Proteins with evolutionarily hypervariable domains are associated with immune response and better survival of basal-like breast cancer patients. Comput Struct Biotechnol J. (2019) 17:430–40. doi: 10.1016/j.csbj.2019.03.008. PMID: 30996822 PMC6451114

[56] Dai L Xia L Su G Gao Y Jiang Q Yang P . Identifying prioritization of therapeutic targets for ankylosing spondylitis: a multi-omics Mendelian randomization study. J Transl Med. (2024) 22:1115. doi: 10.1186/s12967-024-05925-x. PMID: 39707330 PMC11662797

[57] Nakagawa T Roth W Wong P Nelson A Farr A Deussing J . Cathepsin L: critical role in Ii degradation and CD4 T cell selection in the thymus. Science. (1998) 280:450–3. doi: 10.1126/science.280.5362.450. PMID: 9545226

[58] Hageman I Mol F Atiqi S Joustra V Sengul H Henneman P . Novel DNA methylome biomarkers associated with adalimumab response in rheumatoid arthritis patients. Front Immunol. (2023) 14:1303231. doi: 10.3389/fimmu.2023.1303231. PMID: 38187379 PMC10771853

[59] da Rocha LF Duarte ÂLBP Dantas AT Mariz HA Pitta IdR Galdino SL . Increased serum interleukin 22 in patients with rheumatoid arthritis and correlation with disease activity. J Rheumatol. (2012) 39:1320–5. doi: 10.3899/jrheum.111027. PMID: 22589261

[60] Xie Q Wang SC Li J . Interleukin 22, a potential therapeutic target for rheumatoid arthritis. J Rheumatol. (2012) 39:2220. doi: 10.3899/jrheum.120757. PMID: 23118293

[61] Slouma M Kharrat L Tezegdenti A Metoui L Dhahri R Ghazouani E . Increased serum interleukin 22 levels in patients with axial spondyloarthritis. Expert Rev Clin Immunol. (2023) 19:123–9. doi: 10.1080/1744666x.2023.2142563. PMID: 36326666

[62] Ikeuchi H Kuroiwa T Hiramatsu N Kaneko Y Hiromura K Ueki K . Expression of interleukin-22 in rheumatoid arthritis: potential role as a proinflammatory cytokine. Arthritis Rheum. (2005) 52:1037–46. doi: 10.1002/art.20965. PMID: 15818686

[63] Ciccia F Accardo-Palumbo A Alessandro R Rizzo A Principe S Peralta S . Interleukin-22 and interleukin-22-producing NKp44+ natural killer cells in subclinical gut inflammation in ankylosing spondylitis. Arthritis Rheum. (2012) 64:1869–78. doi: 10.1097/bor.0000000000000239. PMID: 22213179

[64] Sagiv M Slobodin G Khatib T Rosner I Rozenbaum M Peri R . Ab0119 serum levels of interleukin-22 are high in ankylosing spondylitis, particularly in smokers, but do not correlate with radiographic bone formation nor with disease activity. Ann Rheum Dis. (2020) 79:1359–60. doi: 10.1136/annrheumdis-2020-eular.2961. PMID: 41926762

[65] Funes SC Rios M Fernández-Fierro A Covián C Bueno SM . Naturally derived heme-oxygenase 1 inducers and their therapeutic application to immune-mediated diseases. Front Immunol. (2020) 11:1467. doi: 10.3389/fimmu.2020.01467. PMID: 32849503 PMC7396584

[66] Kobayashi H Takeno M Saito T Takeda Y Kirino Y Noyori K . Regulatory role of heme oxygenase 1 in inflammation of rheumatoid arthritis. Arthritis Rheum. (2006) 54:1132–42. doi: 10.1002/art.21754. PMID: 16572448

