# Supplementary material for: Identification of new overlapping and disease-specific genetic risk factors for rheumatoid arthritis and radiographic axial spondyloarthritis: a meta-analysis of three large European populations and functional characterization
Source: Front Immunol. 2026 Apr 23;17:1637735. doi: 10.3389/fimmu.2026.1637735 (PMC13149237; doi:10.3389/fimmu.2026.1637735)
Supplement: Supplementary file 1 [file Table1.docx]

**Supplementary Table 1.** Linkage disequilibrium between prioritized chromosome 6 variants and classical HLA risk proxies

| **Lead SNP** | **Gene** | **HLA proxy SNP** | **Tagged HLA allele** | **r² (EUR)** |
| --- | --- | --- | --- | --- |
| rs1977199 | BTN2A1 | rs4349859 | HLA-B27 | 0.0007 |
| rs1977199 | BTN2A1 | rs660895 | HLA-DRB1 | 0.0021 |
| rs9393716 | BTN3A2 | rs4349859 | HLA-B27 | 0.0003 |
| rs9393716 | BTN3A2 | rs660895 | HLA-DRB1 | 0.0001 |

Linkage disequilibrium (LD) between prioritized lead SNPs located on chromosome 6 and proxies of classical HLA risk alleles was evaluated using European populations from the 1000 Genomes Project Phase 3 reference panel. Proxies included rs4349859 (tagging HLA-B27) and rs660895 (tagging HLA-DRB1 risk haplotypes). LD was calculated using a 500-kb window. r² values <0.01 indicate absence of meaningful LD.
