# Supplementary material for: Identification of new overlapping and disease-specific genetic risk factors for rheumatoid arthritis and radiographic axial spondyloarthritis: a meta-analysis of three large European populations and functional characterization
Source: Front Immunol. 2026 Apr 23;17:1637735. doi: 10.3389/fimmu.2026.1637735 (PMC13149237; doi:10.3389/fimmu.2026.1637735)
Supplement: Supplementary file 2 [file Table2.docx]

**Supplementary Table 2.** Association analysis of the 11 SNPs related with risk to develop IMID (RA and AS) in the Discovery populations (UKBB and FinnGen).

| **RA Discovery Population** | | | | | | | | | | |
| --- | --- | --- | --- | --- | --- | --- | --- | --- | --- | --- |
| **SNP** | **Chr.** | **Nearest Gene** | **Minor allele** | **UKBB**  **N= 367,942**  **(Cases=4,380 and controls=363,562)** | | **FinnGen**  **N=** **153,457**  **(Cases=6,236 and controls=147,221)** | | **Meta-analysis**  **N=521,399**  **(Cases=10,616 and controls=510,783)** | | |
|  |  |  |  | **OR (95% CI)** | ***P*** | **OR (95% CI)** | ***P*** | **OR (95% CI)** | ***P*** | ***P_Het_*** |
| rs363075 | 4 | *HTT* | A | 1.12 (1.02-1.22) | 0.015 | 1.13 (1.02-1.25) | 0.015 | **1.12 (1.05-1.20)** | **5.59×10^-04^** | 0.857 |
| rs1977199 | 6 | *BTN2A1* | A | 0.94 (0.90-0.99) | 0.026 | **0.92 (0.87-0.96)** | **3.74×10^-04^** | **0.93 (0.90-0.96)** | **3.67×10^-05^** | 0.372 |
| rs6901425 | 6 | *ZNF322* | G | 0.96 (0.90-1.02) | 0.210 | **0.88 (0.84-0.93)** | **3.00×10^-06^** | **0.91 (0.87-0.95)** | **7.74×10^-06^** | 0.055 |
| rs9393716 | 6 | *BTN3A2* | G | **1.10 (1.04-1.16)** | **4.00×10^-04^** | **1.08 (1.03-1.14)** | **3.01×10^-03^** | **1.09 (1.05-1.13)** | **4.12×10^-06^** | 0.616 |
| rs12718261 | 7 | *IKZF1* | A | 1.06 (1.01-1.10) | 0.022 | **1.07 (1.02-1.10)** | **3.12×10^-03^** | **1.06 (1.03-1.09)** | **1.61×10^-04^** | 0.865 |
| rs66462181 | 6 | *H2BC11* | C | 1.11 (1.03-1.18) | 4.00×10^-03^ | 1.12 (1.02-1.23) | 0.018 | **1.11 (1.05-1.17)** | **2.13×10^-04^** | 0.815 |
| rs72831267 | 6 | *CARMIL1* | C | 0.95 (0.90-0.99) | 0.020 | **0.93 (0.89-0.97)** | **7.99×10^-04^** | **0.94 (0.91-0.97)** | **4.43×10^-05^** | 0.579 |
| rs72843633 | 6 | *PRSS16* | T | 0.98 (0.91-1.05) | 0.550 | **0.89 (0.85-0.94)** | **3.40×10^-05^** | **0.92 (0.88-0.96)** | **2.03×10^-04^** | 0.054 |
| rs72920280 | 6 | *MANEA* | T | **1.13 (1.06-1.21)** | **3.30×10^-04^** | 1.08 (1.02-1.14) | 0.013 | **1.10 (1.05-1-15)** | **2.89×10^-05^** | 0.296 |
| rs73158426 | 7 | *MGAM2* | G | 1.14 (1.02-1.27) | 0.017 | 1.22 (1.06-1.40) | 5.35**×**10^-03^ | **1.17 (1.07-1.27)** | **3.14×10^-04^** | 0.456 |
| rs77601296 | 6 | *ITPR3* | A | 0.93 (0.86-1-00) | 0.042 | **0.89 (0.84-0.94)** | **2.70×10^-05^** | **0.90 (0.86-0.94)** | **5.58×10^-06^** | 0.313 |
| rs2495964 | 6 | *GRM4* | G | 0.97 (0.92-1.01) | 0.150 | **0.90 (0.86-0.95)** | **5.23×10^-06^** | **0.94 (0.91-0.96)** | **1.33×10^-05^** | 0.049 |
| rs9469540 | 6 | *ITPR3* | T | 0.91 (0.87-0.95) | **3.70×10^-05^** | **0.91 (0.88-0.96)** | **5.82×10^-05^** | **0.91 (0.89-0.94)** | **8.11×10^-09^** | 0.944 |
|  |  |  |  |  |  |  |  |  |  |  |
| **AS Discovery Population** | | | | | | | | | | |
| **SNP** | **Chr.** | **Nearest Gene** | **Minor allele** | **UKBB**  **N=364,179**  **(Cases=617 and controls=363,562)** | | **FinnGen**  **N=166,144**  **(Cases=1462 and controls=164,682)** | | **Meta-analysis**  **N=530,323**  **(Cases=2,079 and controls=528,244)** | | |
|  |  |  |  | **OR (95% CI)** | ***P*** | **OR (95% CI)** | ***P*** | **OR (95% CI)** | ***P*** | ***P_Het_*** |
| rs363075 | 4 | *HTT* | A | 1.21 (0.96-1.53) | 6.30×10^-03^ | **1.39 (1.15-1.68)** | **5.76×10^-04^** | **1.32 (1.24-1.52)** | **2.35×10^-04^** | 0.365 |
| rs1977199 | 6 | *BTN2A1* | A | 1.15 (1.01-1.32) | 4.70×10^-03^ | **1.23 (1.12-1.35)** | **1.25×10^-05^** | **1.20 (1.12-1.30)** | **1.82×10^-06^** | 0.408 |
| rs6901425 | 6 | *ZNF322* | G | 0.91 (0.76-1.09) | 0.100 | **0.74 (0.67-0.82)** | **1.23×10^-08^** | **0.78 (0.72-0.85)** | **4.55×10^-08^** | 0.053 |
| rs9393716 | 6 | *BTN3A2* | G | 0.87 (0.76-1.00) | 0.033 | 0.87 (0.79-0.96) | 4.88×10^-03^ | **0.87 (0.80-0.94)** | **6.01×10^-04^** | 0.991 |
| rs12718261 | 7 | *IKZF1* | A | 1.19 (1.05-1.34) | 0.180 | **1.14 (1.06-1.23)** | **5.16×10^-04^** | **1.15 (1.08-1.23)** | **1.01×10^-05^** | 0.597 |
| rs66462181 | 6 | *H2BC11* | C | 0.82 (0.68-0.98) | 0.150 | 0.78 (0.65-0.93) | 5.29×10^-03^ | **0.80 (0.70-0.90)** | **4.15×10^-04^** | 0.675 |
| rs72831267 | 6 | *CARMIL1* | C | 0.92 (0.82-1.04) | 0.049 | **0.82 (0.75-0.89)** | **9.23×10^-07^** | **0.85 (0.79-0.91)** | **1.47×10^-04^** | 0.107 |
| rs72843633 | 6 | *PRSS16* | T | 0.87 (0.72-1.06) | 0.019 | **0.74 (0.67-0.82)** | **1.02×10^-08^** | **0.77 (0.70-0.84)** | **1.04×10^-08^** | 0.144 |
| rs72920280 | 6 | *MANEA* | T | 1.20 (1.01-1.43) | 0.080 | 1.17 (1.04-1.30) | 6.59×10^-03^ | **1.18 (1.07-1.29)** | **7.32×10^-04^** | 0.805 |
| rs73158426 | 7 | *MGAM2* | G | 1.41 (1.07-1.85) | 0.059 | 1.44 (1.10-1.88) | 7.22×10^-03^ | **1.42 (1.18-1.72)** | **2.94×10^-04^** | 0.898 |
| rs77601296 | 6 | *ITPR3* | A | 0.84 (0.70-1.02) | 0.013 | **0.76 (0.68-0.84)** | **5.50×10^-07^** | **0.78 (0.71-0.85)** | **1.81×10^-07^** | 0.334 |
| rs2495964 | 6 | *GRM4* | G | 0.81 (0.66-0.95) | 4.70×10^-03^ | 0.91 (0.82-0.99) | 0.026 | **0.89 (0.84-0.96)** | **7.23**×10^-04^ | 0.252 |
| rs9469540 | 6 | *ITPR3* | T | 0.87 (0.78-0.97) | 0.013 | **0.83 (0.76-0.90)** | **7.12**×10^-06^ | **0.84 (0.79-0.90)** | **4.23**×10^-07^ | 0.503 |

Abbreviations: SNP, single nucleotide polymorphism; OR, Odds Ratio; CI, Confidence Interval.

*P_Bonferroni correction_*=0.05/15 SNPs= 0.0033.
