# Supplementary material for: Identification of new overlapping and disease-specific genetic risk factors for rheumatoid arthritis and radiographic axial spondyloarthritis: a meta-analysis of three large European populations and functional characterization
Source: Front Immunol. 2026 Apr 23;17:1637735. doi: 10.3389/fimmu.2026.1637735 (PMC13149237; doi:10.3389/fimmu.2026.1637735)
Supplement: Supplementary file 3 [file Table3.docx]

**Supplementary Table 3.** Association analysis of the 11 SNPs in the replication populations (REPAIR cohort).

| **SNP** | **Chr.** | **Nearest Gene** | **Minor allele** | **RA_REPAIR cohort** | |  | **AS_REPAIR cohort** | |
| --- | --- | --- | --- | --- | --- | --- | --- | --- |
|  |  |  |  | **N=4,079**  **(2,044 cases/2,035 controls)** | |  | **N=2,517**  **(367 cases/2,150 controls)** | |
|  |  |  |  | **OR (95% CI)** | ***P*** |  | **OR (95% CI)** | ***P*** |
| rs363075 | 4 | *HTT* | A | 0.99 (0.79-1.24) | 0.917 |  | 1.29 (0.89-1.86) | 0.180 |
| rs1977199 | 6 | *BTN2A1* | A | 0.89 (0.77-1.03) | 0.084 |  | 1.38 (1.16-1.64) | 0.009 |
| rs6901425 | 6 | *ZNF322* | G | 0.84 (0.67-1.05) | 0.134 |  | 1.17 (0.82-1.68) | 0.377 |
| rs9393716 | 6 | *BTN3A2* | G | 0.93 (0.80-1.09) | 0.380 |  | **0.61 (0.47-0.81)** | **4.50×10^-04^** |
| rs12718261 | 7 | *IKZF1* | A | 0.87 (0.77-0.99) | 0.029 |  | 0.95 (0.77-1.10) | 0.639 |
| rs66462181 | 6 | *H2BC11* | C | 1.36 (1.07-1.74) | 0.014 |  | 0.63 (0.38-1.05) | 0.079 |
| rs72831267 | 6 | *CARMIL1* | C | 0.99 (0.87-1.13) | 0.912 |  | 0.99 (0.80-1.24) | 0.964 |
| rs72843633 | 6 | *PRSS16* | T | 0.90 (0.71-1.12) | 0.340 |  | 1.30 (0.86-1.00) | 0.208 |
| rs72920280 | 6 | *MANEA* | T | 0.92 (0.76-1.10) | 0.349 |  | 1.26 (0.93-1.69) | 0.139 |
| rs73158426 | 7 | *MGAM2* | G | 1.06 (0.80-1.42) | 0.679 |  | 1.44 (0.91-2.27) | 0.120 |
| rs77601296 | 6 | *ITPR3* | A | 0.85 (0.70-1.04) | 0.123 |  | 1.00 (0.72-1.40) | 0.978 |
| rs2495964 | 6 | GRM4 | G | 0.86 (0.76-0.97) | 0.016 |  | 0.92 (0.74-1.14) | 0.443 |
| rs9469540 | 6 | ITPR3 | T | 0.90 (0.80-1.02) | 0.091 |  | 1.07 (0.87-1-31) | 0.532 |

Abbreviations: SNP, single nucleotide polymorphism; OR, Odds Ratio; CI, Confidence Interval.

*P_Bonferroni correction_*=0.05/15 SNPs= 0.0033.
