# Supplementary material for: Identification of new overlapping and disease-specific genetic risk factors for rheumatoid arthritis and radiographic axial spondyloarthritis: a meta-analysis of three large European populations and functional characterization
Source: Front Immunol. 2026 Apr 23;17:1637735. doi: 10.3389/fimmu.2026.1637735 (PMC13149237; doi:10.3389/fimmu.2026.1637735)
Supplement: Supplementary file 5 [file Table5.docx]

Supplementary Table 5. Serum and plasma metabolites measured in the HFGP cohort.

| **Serum_markers** | | | | **Plasma_markers** | |
| --- | --- | --- | --- | --- | --- |
| **Panel** | **Assay** | **Uniprot** **ID** **Units** | **OlinkID** | **Assay** | **Units** |
| Olink INFLAMMATION(v.3021) | 4E-BP1 | Q13541 | OID00536 | hsCRP | ug/mL |
| Olink INFLAMMATION(v.3021) | ADA | P00813 | OID00560 | IL18bpx | pg/mL |
| Olink INFLAMMATION(v.3021) | ARTN | Q5T4W7 | OID00526 | Resistin | ng/mL |
| Olink INFLAMMATION(v.3021) | AXIN1 | O15169 | OID00487 | Leptin | ng/mL |
| Olink INFLAMMATION(v.3021) | Beta-NGF | P01138 | OID00519 | Adiponectin | ug/mL |
| Olink INFLAMMATION(v.3021) | CASP-8 | Q14790 | OID00550 | AAT | mg/mL |
| Olink INFLAMMATION(v.3021) | CCL11 | P51671 | OID00505 | IL-1Ra_Q (Quantikine) | pg/mL |
| Olink INFLAMMATION(v.3021) | CCL19 | Q99731 | OID00513 | IL18 pg/mL | pg/mL |
| Olink INFLAMMATION(v.3021) | CCL20 | P78556 | OID00556 | IL-1b pg/mL | pg/mL |
| Olink INFLAMMATION(v.3021)  Olink INFLAMMATION(v.3021) | CCL23  CCL25 | P55773  O15444 | OID00530  OID00551 | IL-6 pg/mL  VEGF-A | pg/mL  pg/mL |
| Olink INFLAMMATION(v.3021) | CCL28 | Q9NRJ3 | OID00539 |  | |
| Olink INFLAMMATION(v.3021) | CCL3 | P10147 | OID00532 |  |  |
| Olink INFLAMMATION(v.3021) | CCL4 | P13236 | OID00498 |  |  |
| Olink INFLAMMATION(v.3021) | CD244 | Q9BZW8 | OID00477 |  |  |
| Olink INFLAMMATION(v.3021) | CD40 | P25942 | OID00542 |  |  |
| Olink INFLAMMATION(v.3021) | CD5 | P06127 | OID00531 |  |  |
| Olink INFLAMMATION(v.3021) | CD6 | P30203 | OID00499 |  |  |
| Olink INFLAMMATION(v.3021) | CD8A | P01732 | OID05124 |  |  |
| Olink INFLAMMATION(v.3021) | CDCP1 | Q9H5V8 | OID00476 |  |  |
| Olink INFLAMMATION(v.3021) | CSF-1 | P09603 | OID00562 |  |  |
| Olink INFLAMMATION(v.3021) | CST5 | P28325 | OID00491 |  |  |
| Olink INFLAMMATION(v.3021) | CX3CL1 | P78423 | OID00552 |  |  |
| Olink INFLAMMATION(v.3021) | CXCL1 | P09341 | OID00496 |  |  |
| Olink INFLAMMATION(v.3021) | CXCL10 | P02778 | OID00535 |  |  |
| Olink INFLAMMATION(v.3021) | CXCL11 | O14625 | OID00486 |  |  |
| Olink INFLAMMATION(v.3021) | CXCL5 | P42830 | OID00520 |  |  |
| Olink INFLAMMATION(v.3021) | CXCL6 | P80162 | OID00534 |  |  |
| Olink INFLAMMATION(v.3021) | CXCL9 | Q07325 | OID00490 |  |  |
| Olink INFLAMMATION(v.3021) | DNER | Q8NFT8 | OID01213 |  |  |
| Olink INFLAMMATION(v.3021) | EN-RAGE | P80511 | OID00541 |  |  |
| Olink INFLAMMATION(v.3021) | FGF-19 | O95750 | OID00545 |  |  |
| Olink INFLAMMATION(v.3021) | FGF-21 | Q9NSA1 | OID00512 |  |  |
| Olink INFLAMMATION(v.3021) | FGF-23 | Q9GZV9 | OID00507 |  |  |
| Olink INFLAMMATION(v.3021) | FGF-5 | P12034 | OID00509 |  |  |
| Olink INFLAMMATION(v.3021) | Flt3L | P49771 | OID00533 |  |  |
| Olink INFLAMMATION(v.3021) | GDNF | P39905 | OID00475 |  |  |
| Olink INFLAMMATION(v.3021) | HGF | P14210 | OID00522 |  |  |
| Olink INFLAMMATION(v.3021) | IFN-gamma | P01579 | OID05547 |  |  |
| Olink INFLAMMATION(v.3021) | IL-1 alpha | P01583 | OID00493 |  |  |
| Olink INFLAMMATION(v.3021) | IL10 | P22301 | OID00528 |  |  |
| Olink INFLAMMATION(v.3021) | IL-10RA | Q13651 | OID00508 |  |  |
| Olink INFLAMMATION(v.3021) | IL-10RB | Q08334 | OID00515 |  |  |
| Olink INFLAMMATION(v.3021) | IL-12B | P29460 | OID00523 |  |  |
| Olink INFLAMMATION(v.3021) | IL13 | P35225 | OID00525 |  |  |
| Olink INFLAMMATION(v.3021) | IL-15RA | Q13261 | OID00514 |  |  |
| Olink INFLAMMATION(v.3021) | IL-17A | Q16552 | OID00485 |  |  |
| Olink INFLAMMATION(v.3021) | IL-17C | Q9P0M4 | OID00483 |  |  |
| Olink INFLAMMATION(v.3021) | IL18 | Q14116 | OID00501 |  |  |
| Olink INFLAMMATION(v.3021) | IL-18R1 | Q13478 | OID00517 |  |  |
| Olink INFLAMMATION(v.3021) | IL2 | P60568 | OID00495 |  |  |
| Olink INFLAMMATION(v.3021) | IL-20 | Q9NYY1 | OID00537 |  |  |
| Olink INFLAMMATION(v.3021) | IL-20RA | Q9UHF4 | OID00489 |  |  |
| Olink INFLAMMATION(v.3021) | IL-22 RA1 | Q8N6P7 | OID00516 |  |  |
| Olink INFLAMMATION(v.3021) | IL-24 | Q13007 | OID00524 |  |  |
| Olink INFLAMMATION(v.3021) | IL-2RB | P14784 | OID00492 |  |  |
| Olink INFLAMMATION(v.3021) | IL33 | O95760 | OID00543 |  |  |
| Olink INFLAMMATION(v.3021) | IL4 | P05112 | OID00546 |  |  |
| Olink INFLAMMATION(v.3021) | IL5 | P05113 | OID00559 |  |  |
| Olink INFLAMMATION(v.3021) | IL6 | P05231 | OID00482 |  |  |
| Olink INFLAMMATION(v.3021) | IL7 | P13232 | OID00478 |  |  |
| Olink INFLAMMATION(v.3021) | IL8 | P10145 | OID00471 |  |  |
| Olink INFLAMMATION(v.3021) | LAP TGF-beta-1 | P01137 | OID00480 |  |  |

| Olink INFLAMMATION(v.3021) | LIF | P15018 | OID00547 |
| --- | --- | --- | --- |
| Olink INFLAMMATION(v.3021) | LIF-R | P42702 | OID00511 |
| Olink INFLAMMATION(v.3021) | MCP-1 | P13500 | OID00484 |
| Olink INFLAMMATION(v.3021) | MCP-2 | P80075 | OID00549 |
| Olink INFLAMMATION(v.3021) | MCP-3 | P80098 | OID00474 |
| Olink INFLAMMATION(v.3021) | MCP-4 | Q99616 | OID00504 |
| Olink INFLAMMATION(v.3021) | MMP-1 | P03956 | OID00510 |
| Olink INFLAMMATION(v.3021) | MMP-10 | P09238 | OID00527 |
| Olink INFLAMMATION(v.3021) | NRTN | Q99748 | OID00548 |
| Olink INFLAMMATION(v.3021) | NT-3 | P20783 | OID00554 |
| Olink INFLAMMATION(v.3021) | OPG | O00300 | OID00479 |
| Olink INFLAMMATION(v.3021) | OSM | P13725 | OID00494 |
| Olink INFLAMMATION(v.3021) | PD-L1 | Q9NZQ7 | OID00518 |
| Olink INFLAMMATION(v.3021) | SCF | P21583 | OID00500 |
| Olink INFLAMMATION(v.3021) | SIRT2 | Q8IXJ6 | OID00538 |
| Olink INFLAMMATION(v.3021) | SLAMF1 | Q13291 | OID00502 |
| Olink INFLAMMATION(v.3021) | ST1A1 | P50225 | OID00557 |
| Olink INFLAMMATION(v.3021) | STAMBP | O95630 | OID00558 |
| Olink INFLAMMATION(v.3021) | TGF-alpha | P01135 | OID00503 |
| Olink INFLAMMATION(v.3021) | TNF | P01375 | OID05548 |
| Olink INFLAMMATION(v.3021) | TNFB | P01374 | OID00561 |
| Olink INFLAMMATION(v.3021) | TNFRSF9 | Q07011 | OID00553 |
| Olink INFLAMMATION(v.3021) | TNFSF14 | O43557 | OID00506 |
| Olink INFLAMMATION(v.3021) | TRAIL | P50591 | OID00488 |
| Olink INFLAMMATION(v.3021) | TRANCE | O14788 | OID00521 |
| Olink INFLAMMATION(v.3021) | TSLP | Q969D9 | OID00497 |
| Olink INFLAMMATION(v.3021) | TWEAK | O43508 | OID00555 |
| Olink INFLAMMATION(v.3021) | uPA | P00749 | OID00481 |
| Olink INFLAMMATION(v.3021) | VEGFA | P15692 | OID00472 |
